# Supplementary material for: Metagenomics reveals novel microbial signatures of farm exposures in house dust
Source: medRxiv. 2023 Apr 12:2023.04.07.23288301. Preprint. [Version 1] doi: 10.1101/2023.04.07.23288301 (PMC10120797; doi:10.1101/2023.04.07.23288301)
Supplement: Supplement 3 [file media-3.pdf]

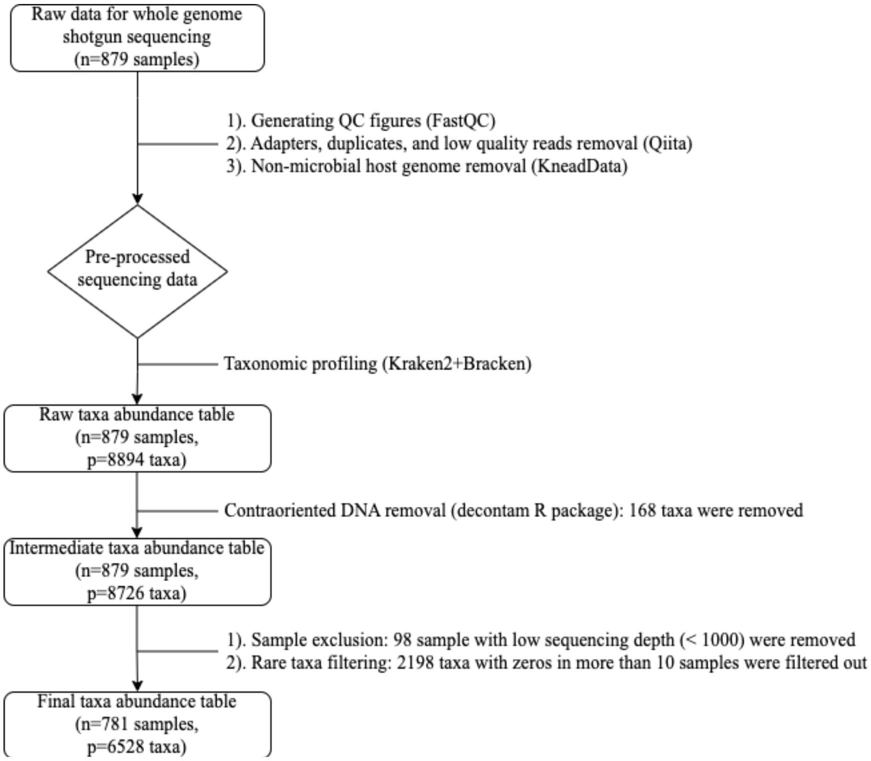

Supplementary Figure 1. Workflow of the Quality Control for WGS.

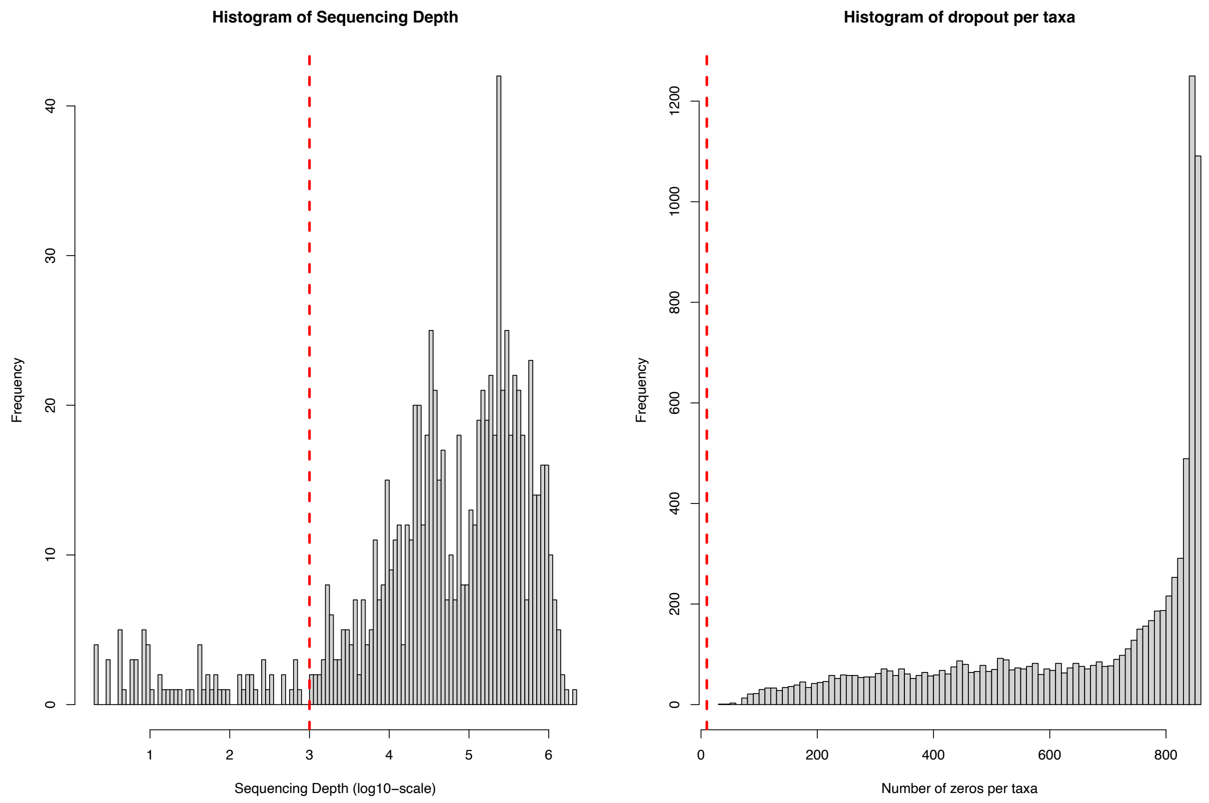

Supplementary Figure 2. Sample and rare taxa filtering criteria for WGS. **(a).** Histogram of sequencing depth. **(b).** Histogram of number of zeros per taxon. Red dotted lines indicate the threshold.

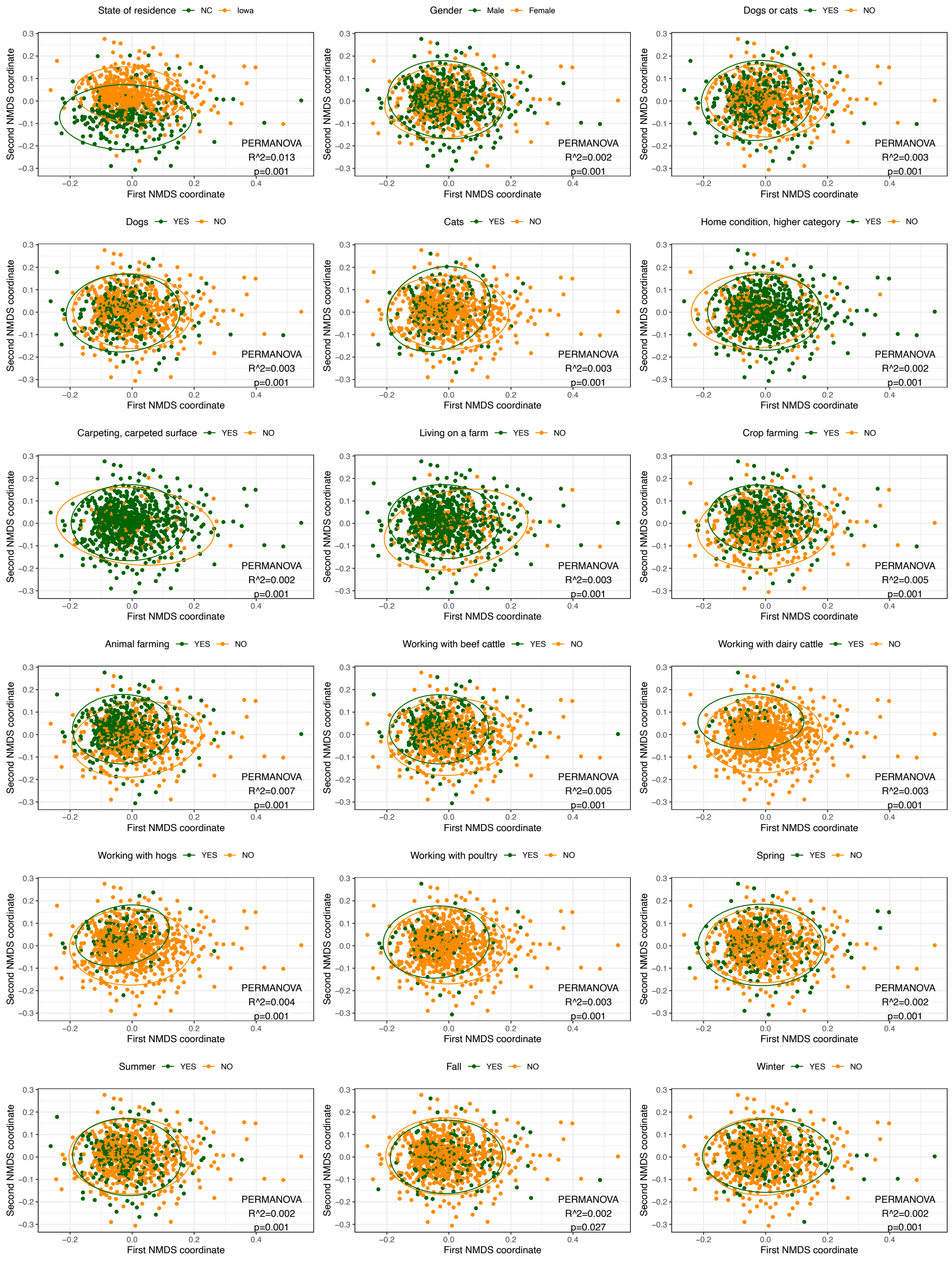

Supplementary Figure 3. Non-metric multidimensional scaling (NMDS) analysis based on unweighted UniFrac distances for all exposures. The dust microbial community of each sample is indicated with one dot. R^2 value (percentage of variance explained by exposure) and p-value from the PERMANOVA analysis are reported.

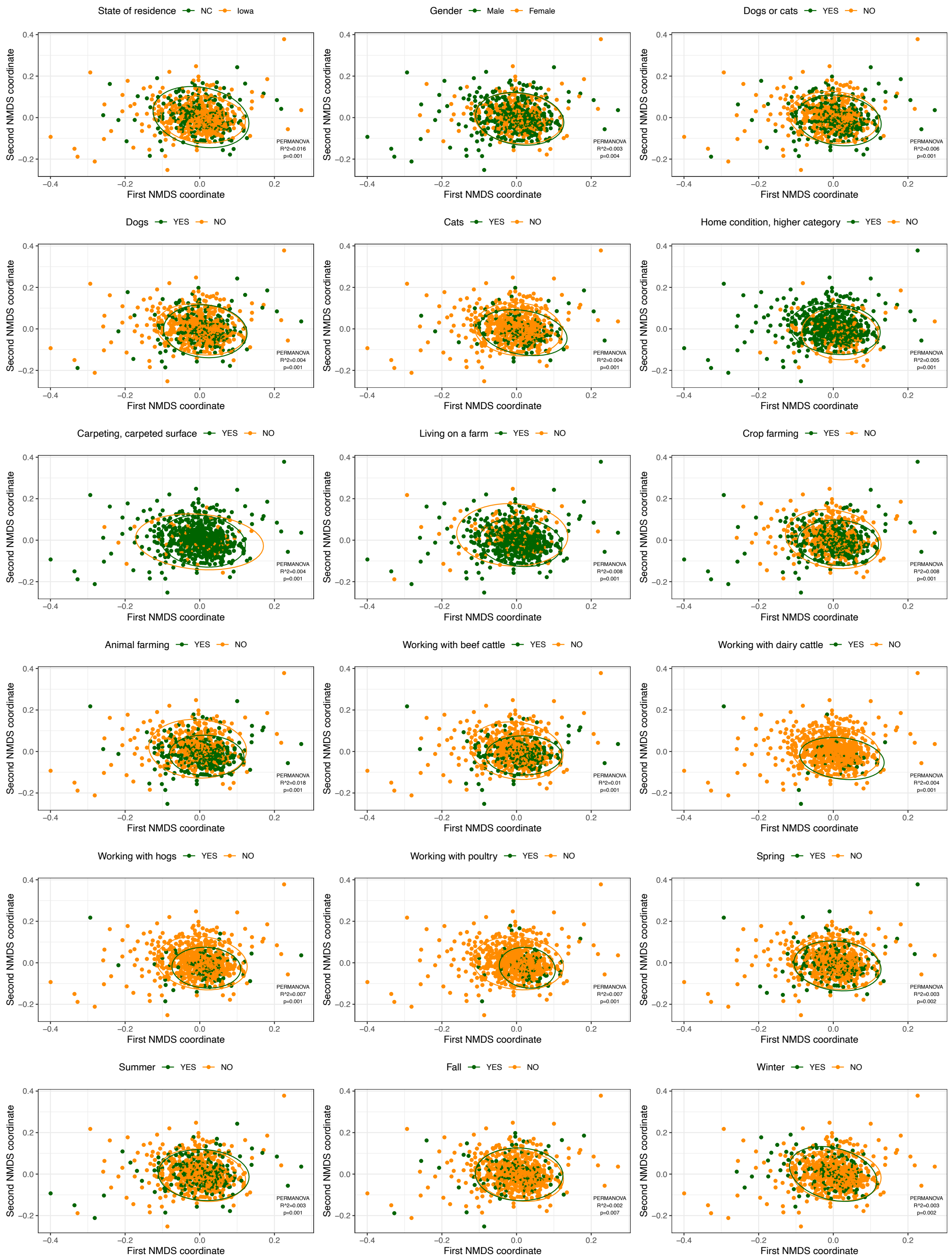

Supplementary Figure 4. Non-metric multidimensional scaling (NMDS) analysis based on weighted UniFrac distances for all exposures. The dust microbial community of each sample is indicated with one dot. R^2 value (percentage of variance explained by exposure) and p-value from the PERMANOVA analysis are reported.
