# Supplementary material for: Metagenomics reveals novel microbial signatures of farm exposures in house dust
Source: medRxiv. 2023 Apr 12:2023.04.07.23288301. Preprint. [Version 1] doi: 10.1101/2023.04.07.23288301 (PMC10120797; doi:10.1101/2023.04.07.23288301)
Supplement: Supplement 5 [file media-5.pdf]

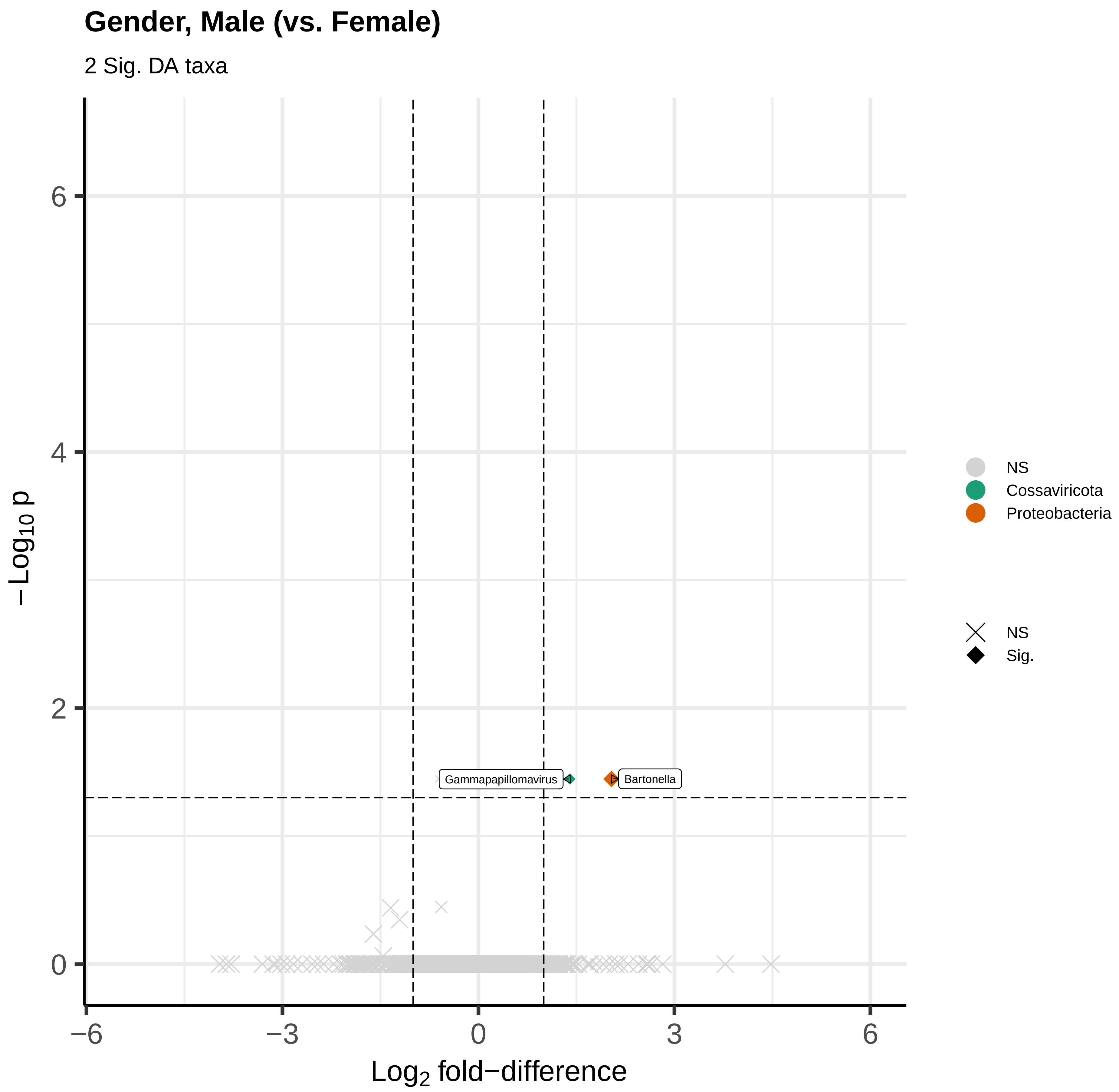
Gender, Male (vs. Female)
2 Sig. DA taxa
Gammapapillomavirus
Bartonella
−Log10 p
Log2 fold−difference
−6
−3
0
3
6
0
2
4
6
NS
Cossaviricota
Proteobacteria
NS
Sig.

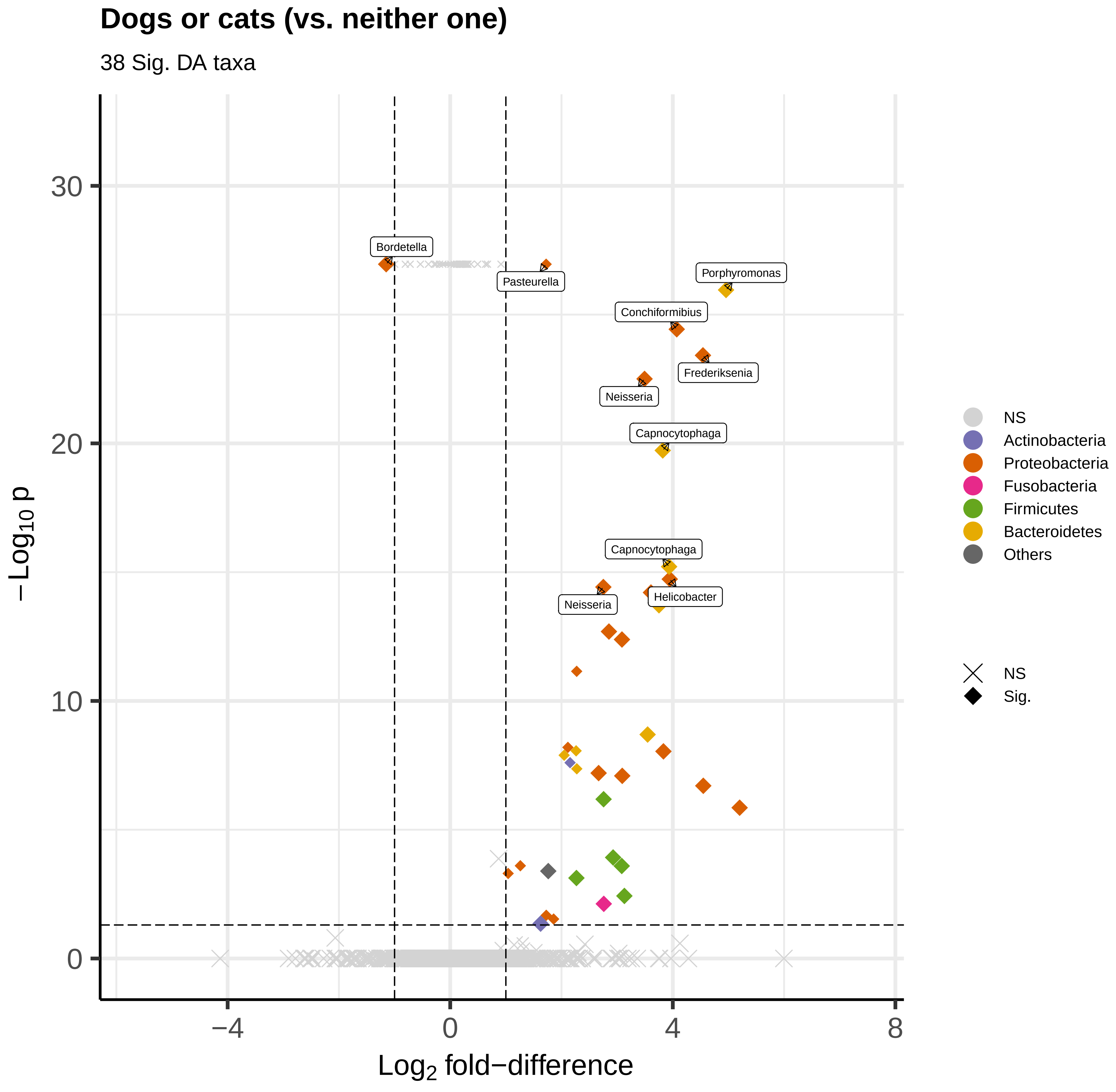

Dogs or cats (vs. neither one)
38 Sig. DA taxa
Bordetella
Pasteurella
Porphyromonas
Conchiformibius
Frederiksenia
Neisseria
Capnocytophaga
Capnocytophaga
Neisseria
Helicobacter
30
20
10
0
−4
0
4
8
$-Log_{10}$ p
Log_2 fold−difference
NS
Actinobacteria
Proteobacteria
Fusobacteria
Firmicutes
Bacteroidetes
Others
NS
Sig.

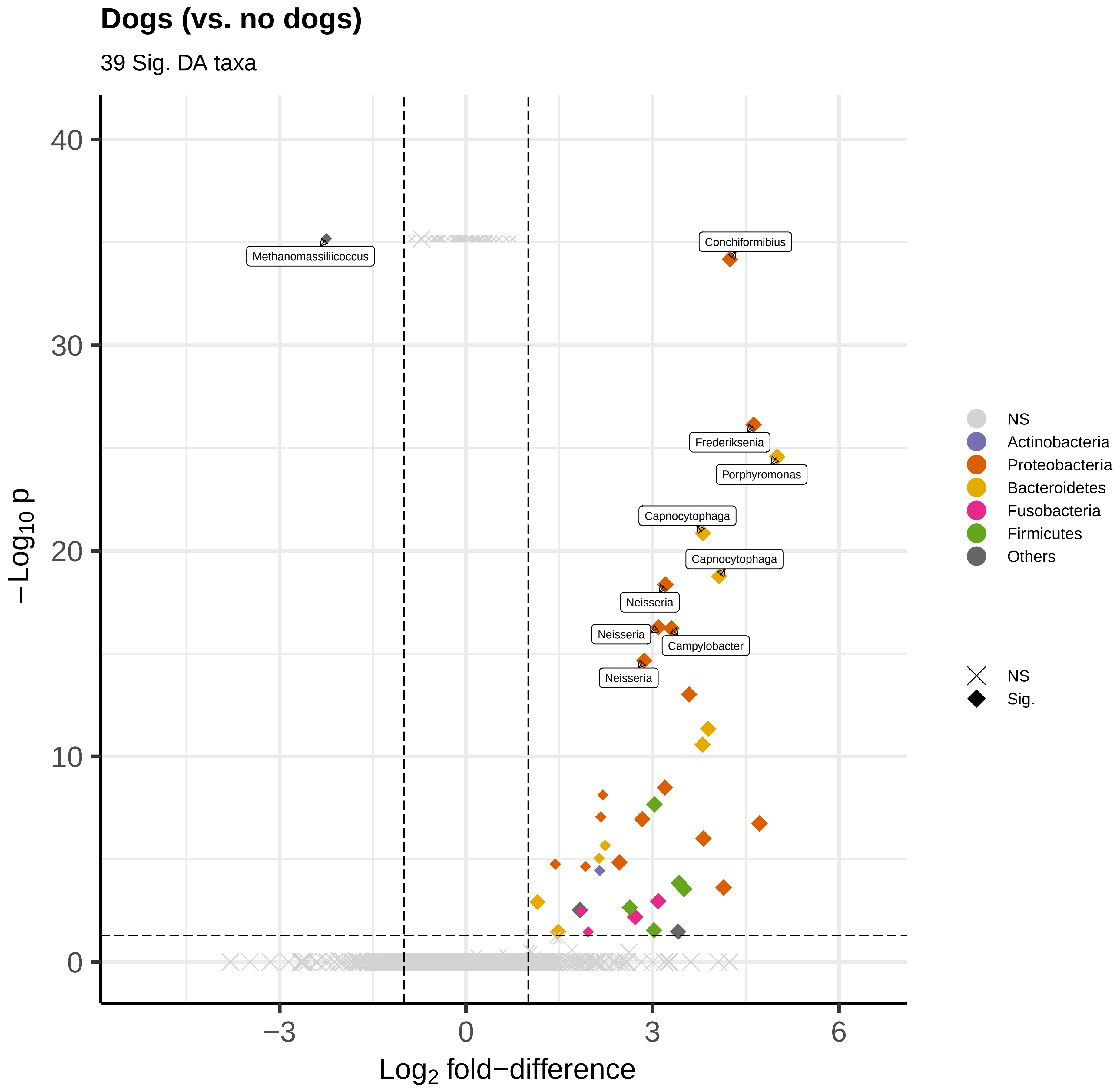

Dogs (vs. no dogs)
39 Sig. DA taxa
Methanomassiliicoccus
Conchiformibius
Frederiksenia
Porphyromonas
Capnocytophaga
Capnocytophaga
Neisseria
Neisseria
Campylobacter
Neisseria
40
30
20
10
0
−3
0
3
6
$-\log_{10}$ p
$\log_2$ fold−difference
NS
Actinobacteria
Proteobacteria
Bacteroidetes
Fusobacteria
Firmicutes
Others
NS
Sig.

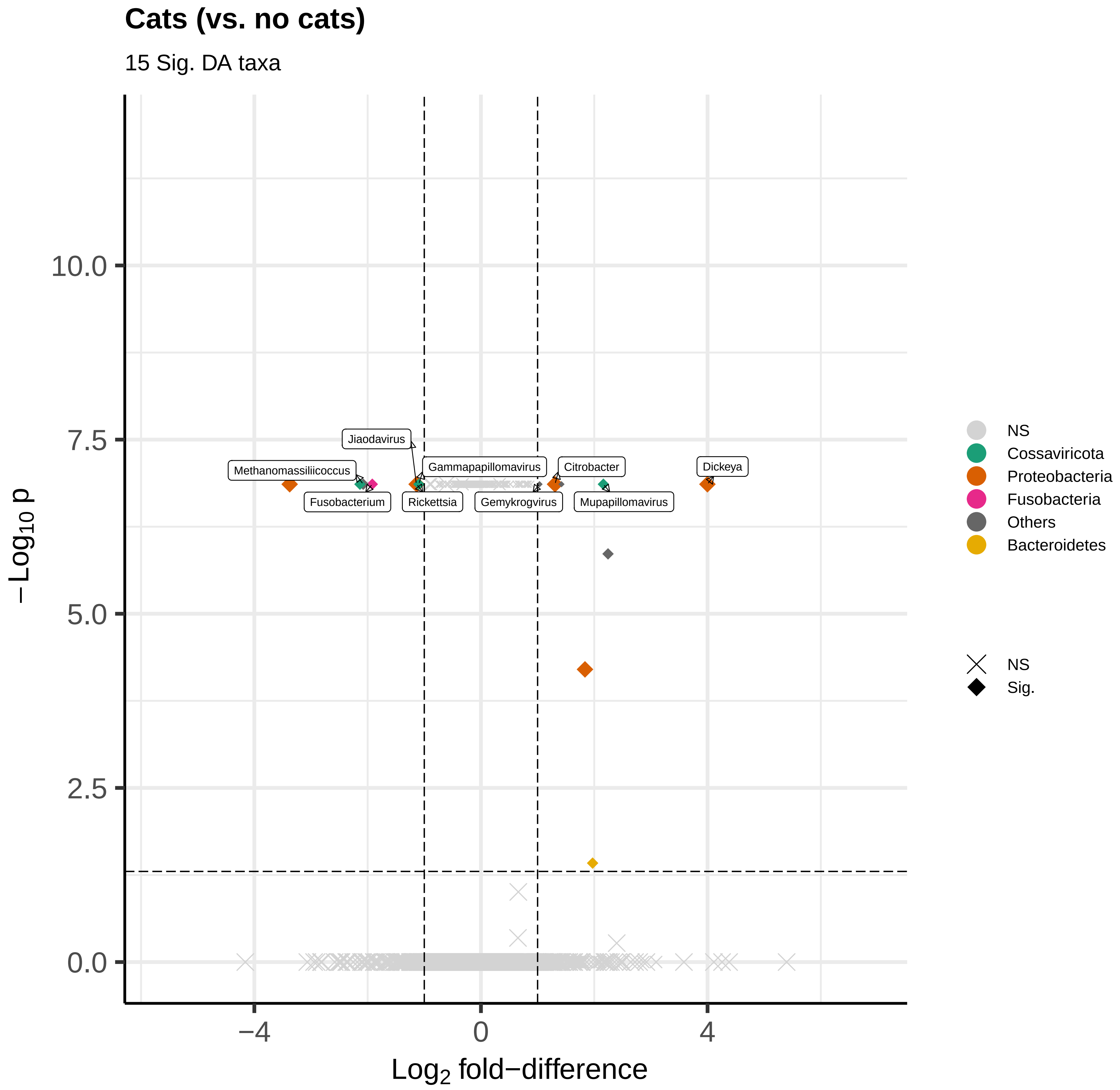
Cats (vs. no cats)
15 Sig. DA taxa
−Log10 p
Log2 fold−difference
10.0
7.5
5.0
2.5
0.0
−4
0
4
Jiaodavirus
Methanomassiliicoccus
Gammapapillomavirus
Citrobacter
Dickeya
Fusobacterium
Rickettsia
Gemykrogvirus
Mupapillomavirus
NS
Cossaviricota
Proteobacteria
Fusobacteria
Others
Bacteroidetes
NS
Sig.

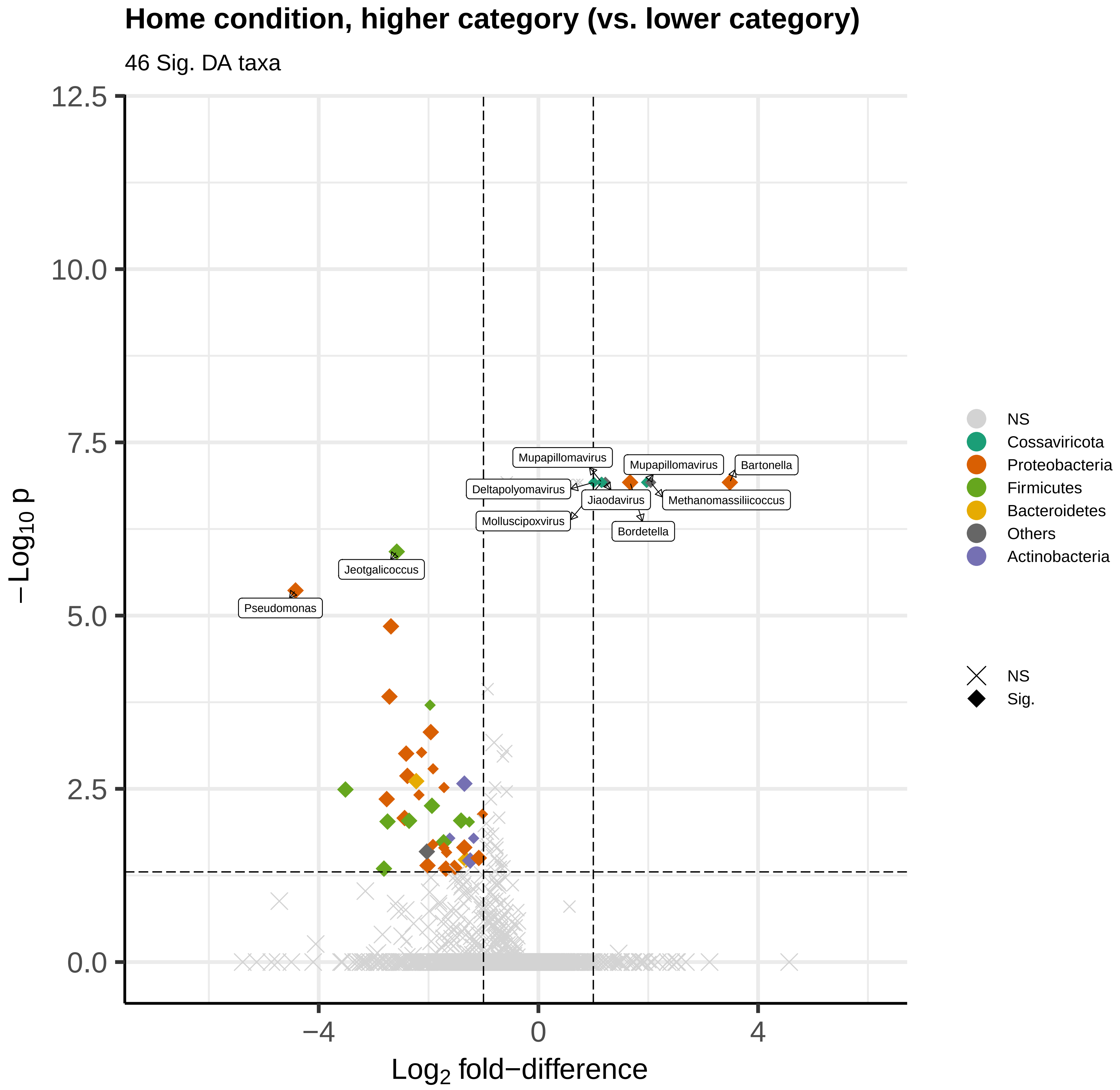

Home condition, higher category (vs. lower category)
46 Sig. DA taxa
12.5
10.0
7.5
5.0
2.5
0.0
−4
0
4
$-\text{Log}_{10}$ p
Log_2 fold−difference
Mupapillomavirus
Mupapillomavirus
Bartonella
Deltapolyomavirus
Jiaodavirus
Methanomassiliicoccus
Molluscipoxvirus
Bordetella
Jeotgalicoccus
Pseudomonas
NS
Cossaviricota
Proteobacteria
Firmicutes
Bacteroidetes
Others
Actinobacteria
NS
Sig.

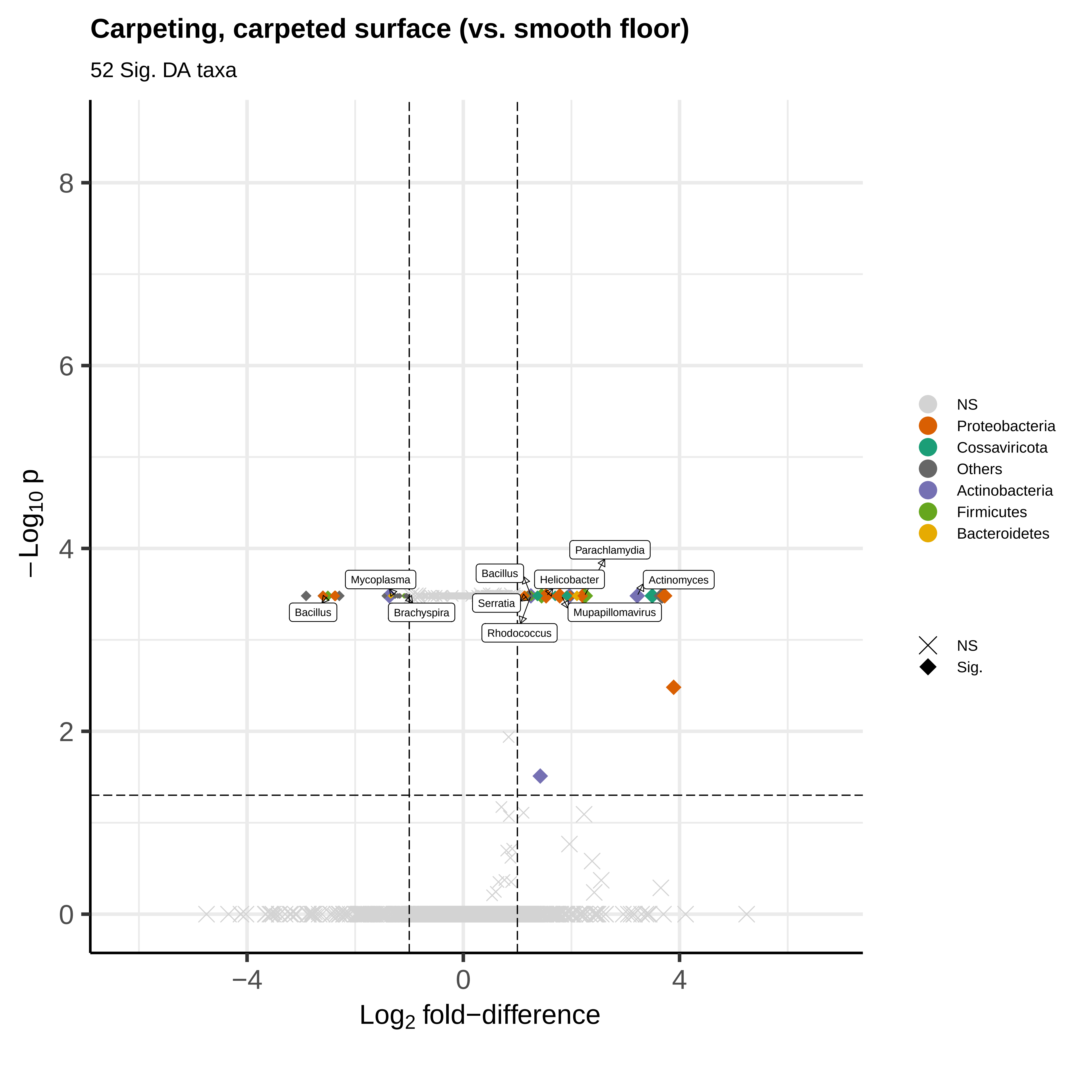

Carpeting, carpeted surface (vs. smooth floor)
52 Sig. DA taxa
−Log10 p
Log2 fold−difference
8
6
4
2
0
−4
0
4
Parachlamydia
Mycoplasma
Bacillus
Helicobacter
Actinomyces
Serratia
Bacillus
Brachyspira
Mupapillomavirus
Rhodococcus
NS
Proteobacteria
Cossaviricota
Others
Actinobacteria
Firmicutes
Bacteroidetes
NS
Sig.

Living on a farm (vs. not living on a farm)

10 Sig. DA taxa

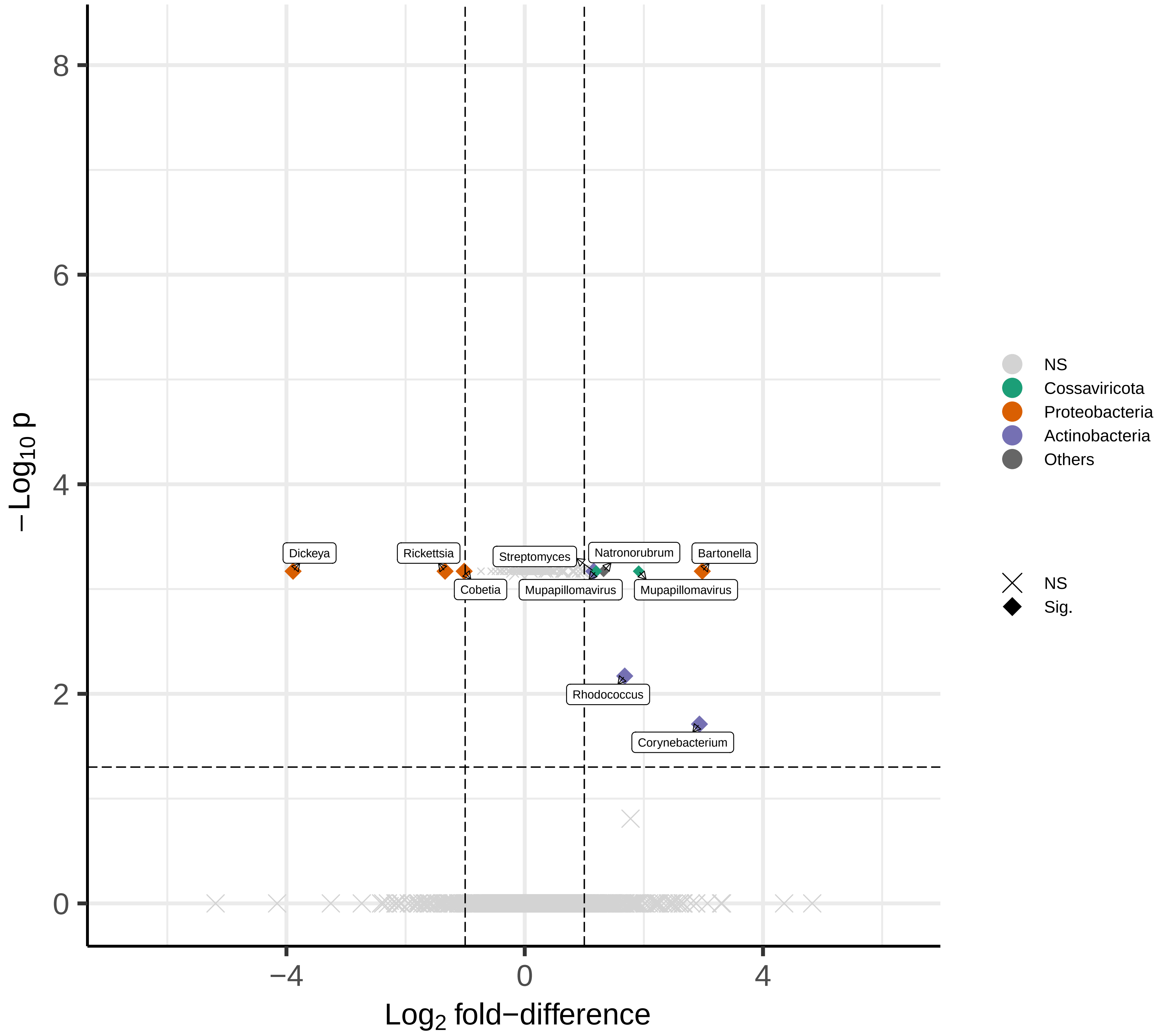

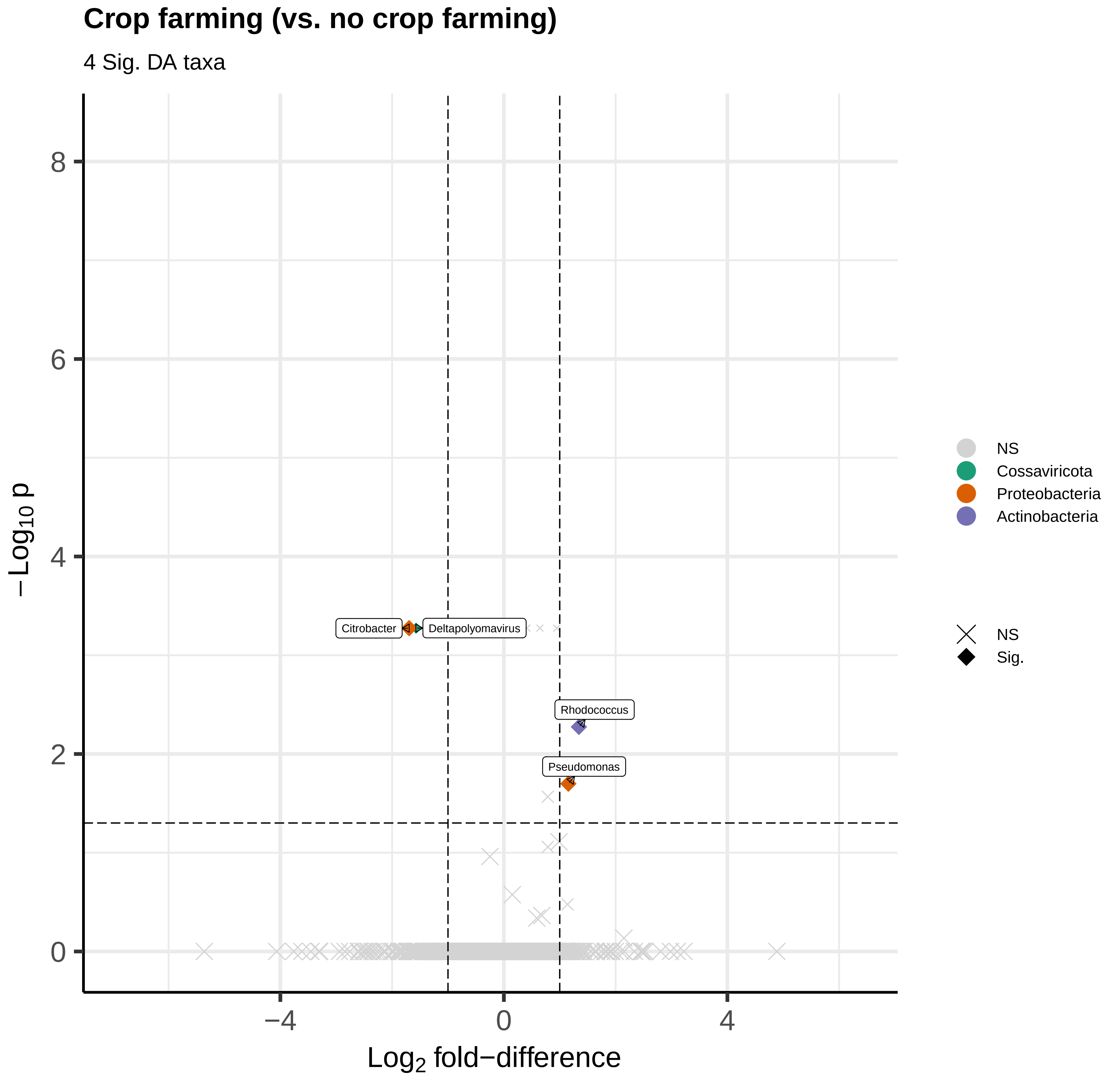

Crop farming (vs. no crop farming)
4 Sig. DA taxa
Citrobacter
Deltapolyomavirus
Rhodococcus
Pseudomonas
8
6
4
2
0
−4
0
4
−Log10 p
Log2 fold−difference
NS
Cossaviricota
Proteobacteria
Actinobacteria
NS
Sig.

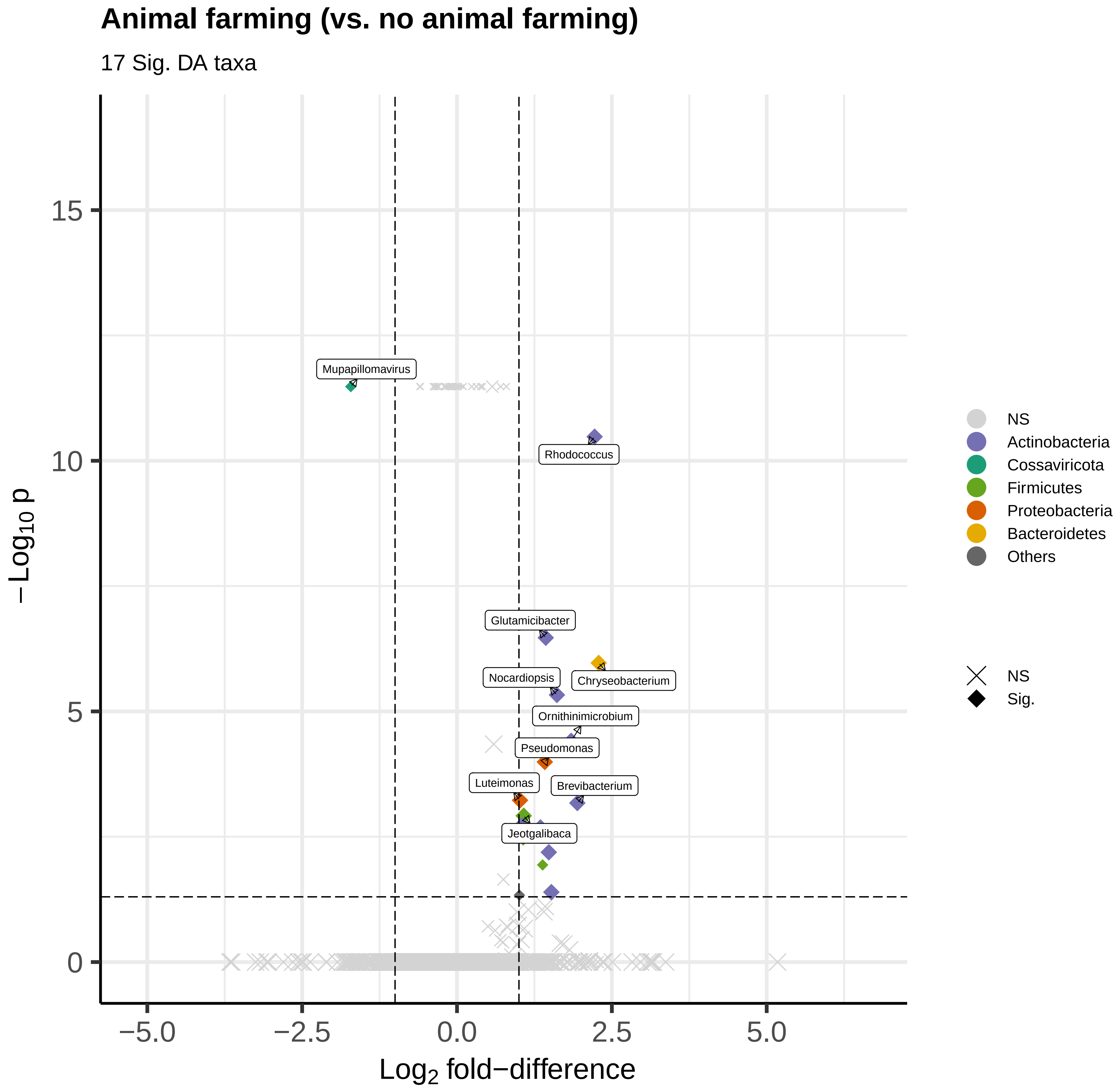

Animal farming (vs. no animal farming)
17 Sig. DA taxa
Mupapillomavirus
Rhodococcus
Glutamicibacter
Nocardiopsis
Chryseobacterium
Ornithinimicrobium
Pseudomonas
Luteimonas
Brevibacterium
Jeotgalibaca
15
10
5
0
−Log10 p
−5.0
−2.5
0.0
2.5
5.0
Log2 fold−difference
NS
Actinobacteria
Cossaviricota
Firmicutes
Proteobacteria
Bacteroidetes
Others
NS
Sig.

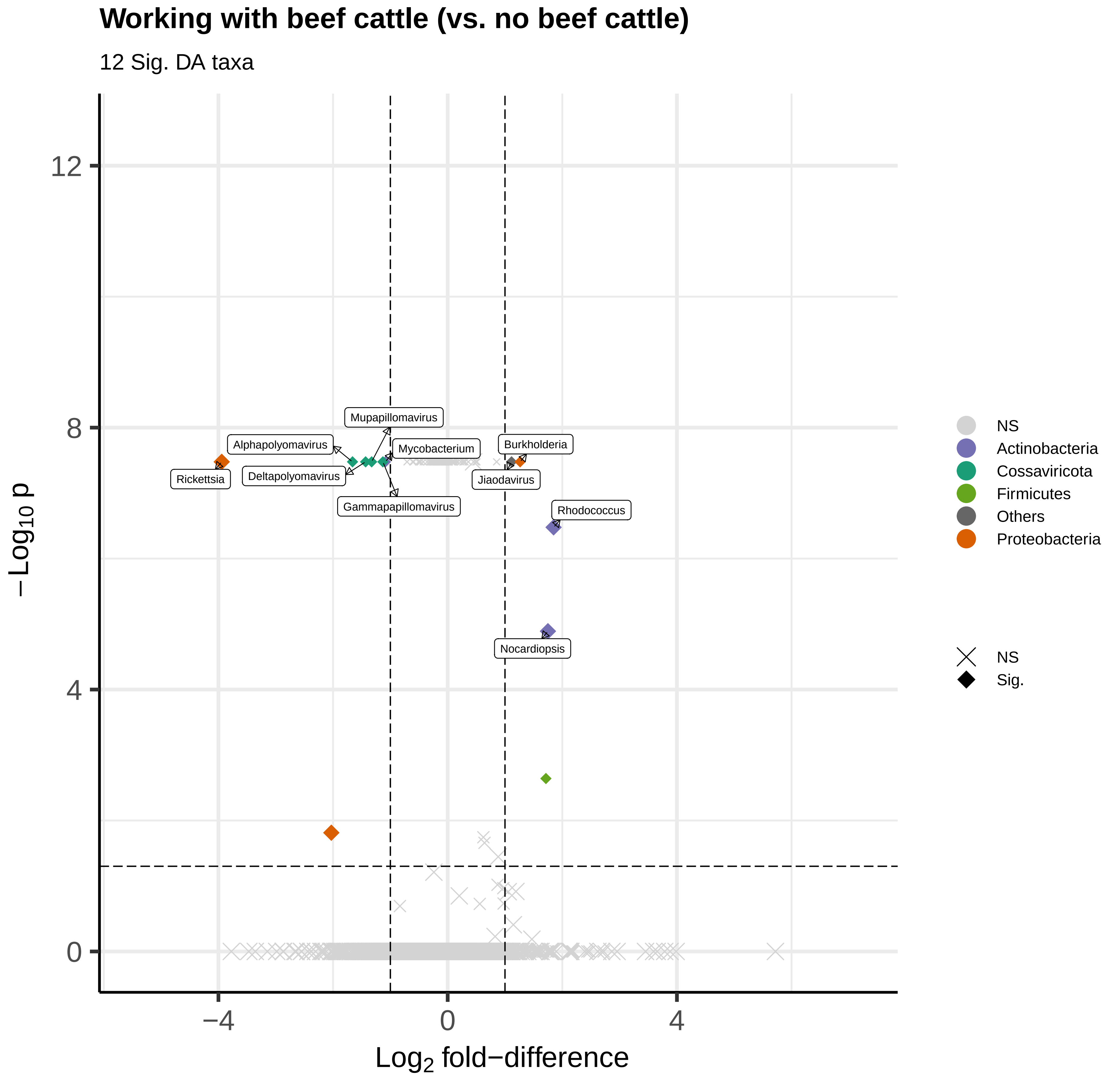

Working with beef cattle (vs. no beef cattle)
12 Sig. DA taxa
Mupapillomavirus
Alphapolyomavirus
Mycobacterium
Burkholderia
Rickettsia
Deltapolyomavirus
Jiaodavirus
Gammapapillomavirus
Rhodococcus
Nocardiopsis
12
8
4
0
−4
0
4
−Log10 p
Log2 fold−difference
NS
Actinobacteria
Cossaviricota
Firmicutes
Others
Proteobacteria
NS
Sig.

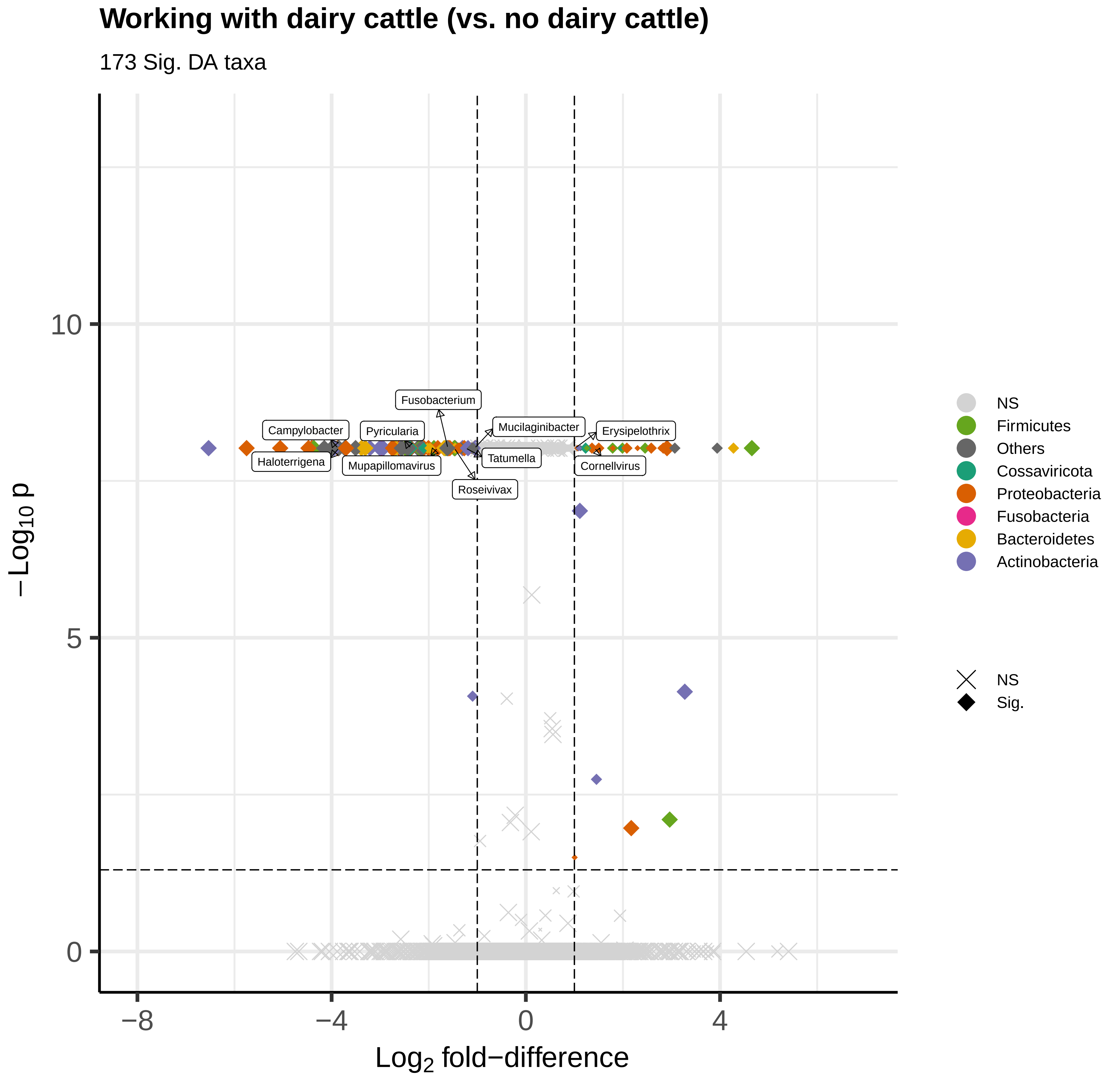

Working with dairy cattle (vs. no dairy cattle)
173 Sig. DA taxa
Fusobacterium
Campylobacter
Pyricularia
Mucilaginibacter
Erysipelothrix
Haloterrigena
Mupapillomavirus
Tatumella
Cornellvirus
Roseivivax
−Log10 p
Log2 fold−difference
NS
Firmicutes
Others
Cossaviricota
Proteobacteria
Fusobacteria
Bacteroidetes
Actinobacteria
NS
Sig.

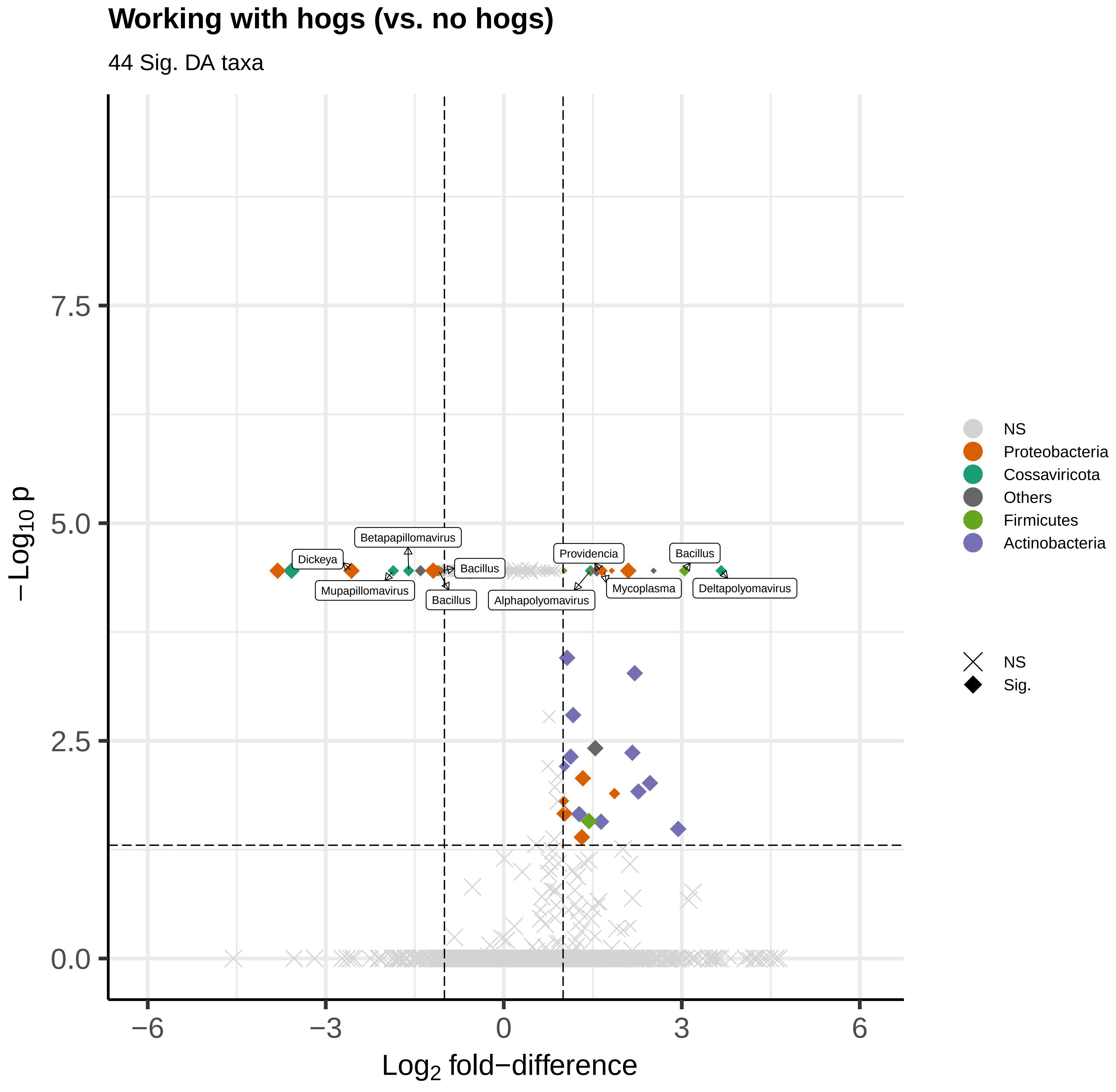

Working with hogs (vs. no hogs)
44 Sig. DA taxa
Betapapillomavirus
Dickeya
Bacillus
Mupapillomavirus
Bacillus
Providencia
Bacillus
Alphapolyomavirus
Mycoplasma
Deltapolyomavirus
−Log10 p
7.5
5.0
2.5
0.0
−6
−3
0
3
6
Log2 fold−difference
NS
Proteobacteria
Cossaviricota
Others
Firmicutes
Actinobacteria
NS
Sig.

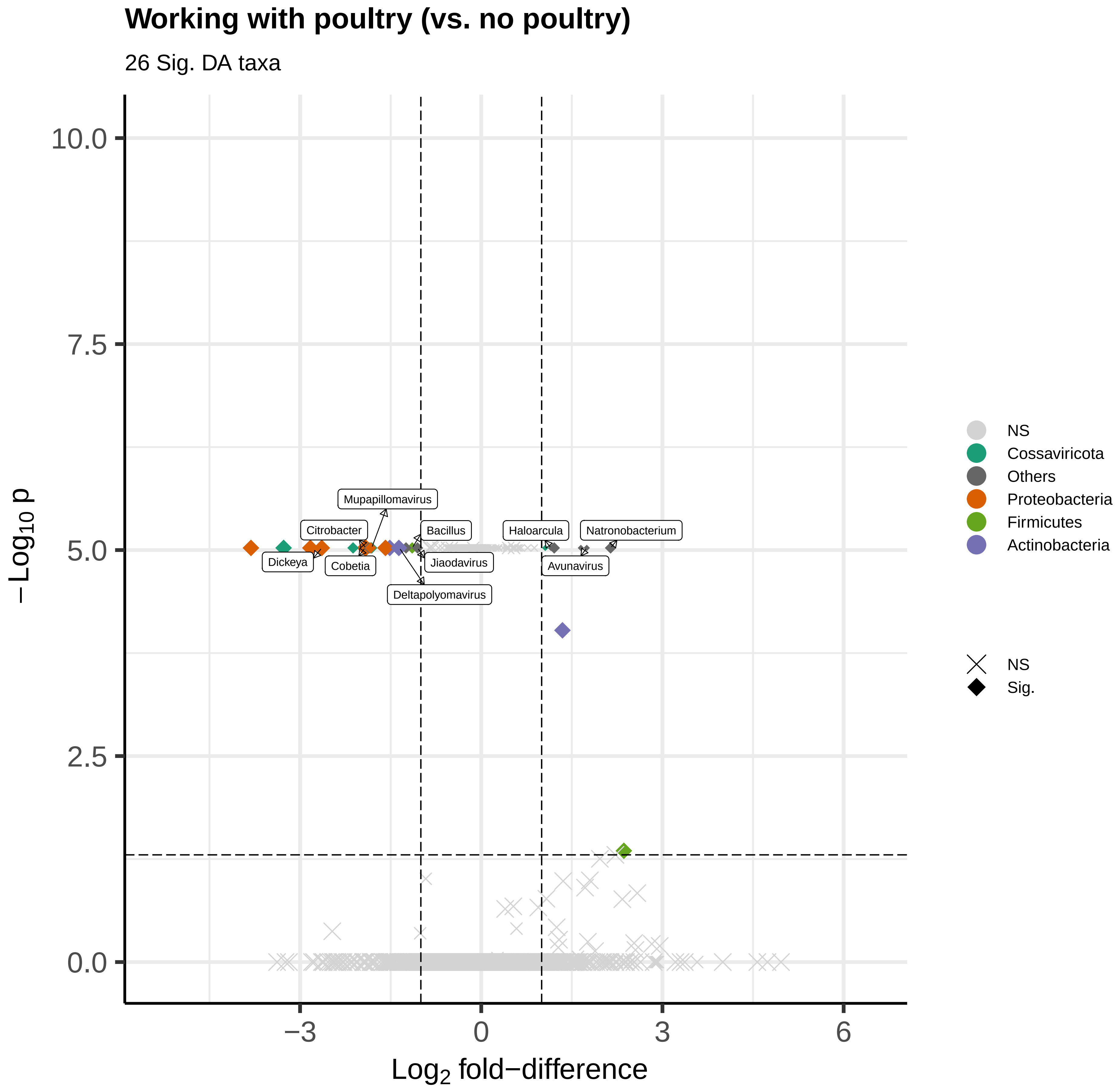

Working with poultry (vs. no poultry)
26 Sig. DA taxa
−Log10 p
10.0
7.5
5.0
2.5
0.0
Mupapillomavirus
Citrobacter
Bacillus
Haloarcula
Natronobacterium
Dickeya
Cobetia
Jiaodavirus
Avunavirus
Deltapolyomavirus
−3
0
3
6
Log2 fold−difference
NS
Cossaviricota
Others
Proteobacteria
Firmicutes
Actinobacteria
NS
Sig.

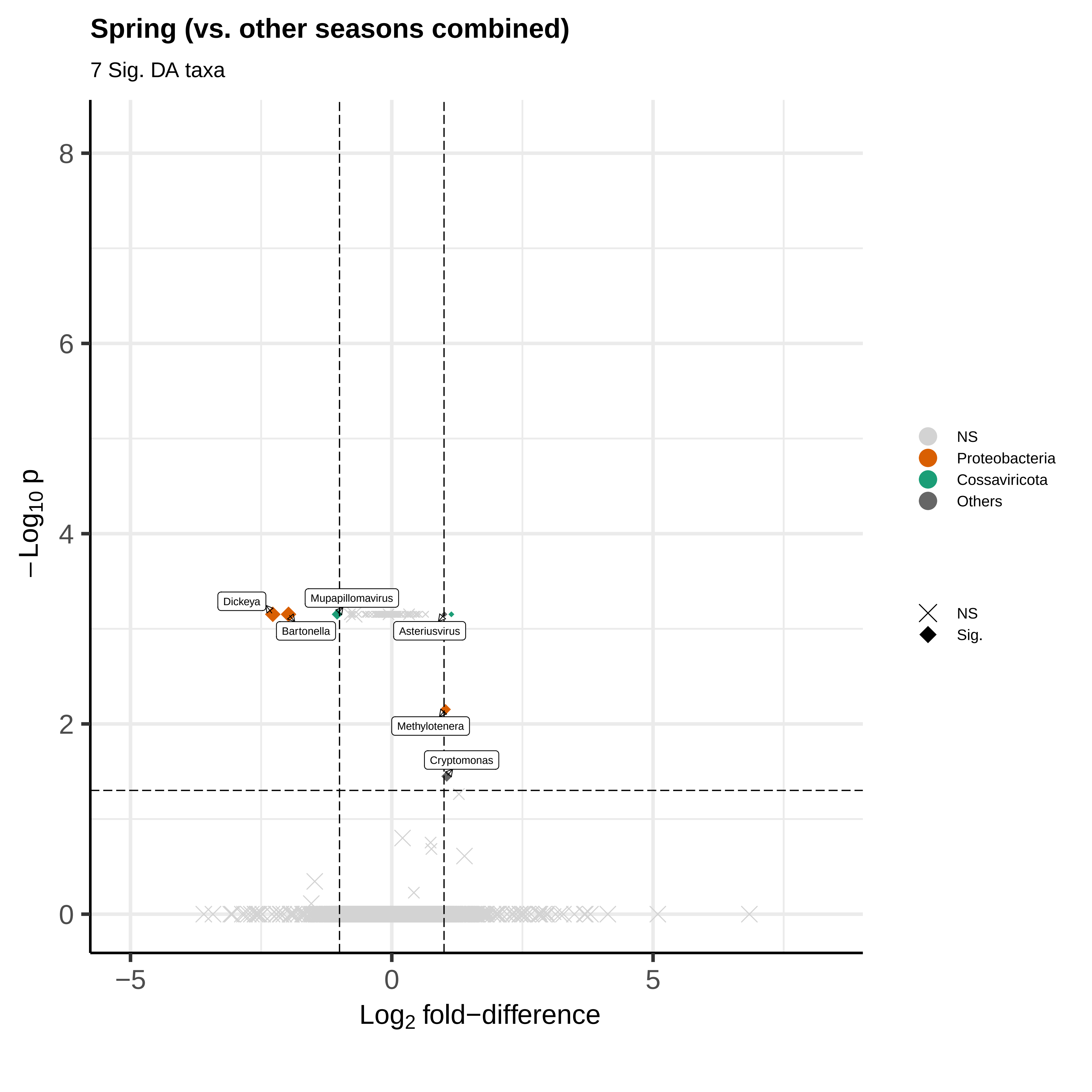
Spring (vs. other seasons combined)
7 Sig. DA taxa
−Log10 p
Log2 fold−difference
8
6
4
2
0
−5
0
5
Dickeya
Bartonella
Mupapillomavirus
Asteriusvirus
Methylotenera
Cryptomonas
NS
Proteobacteria
Cossaviricota
Others
NS
Sig.

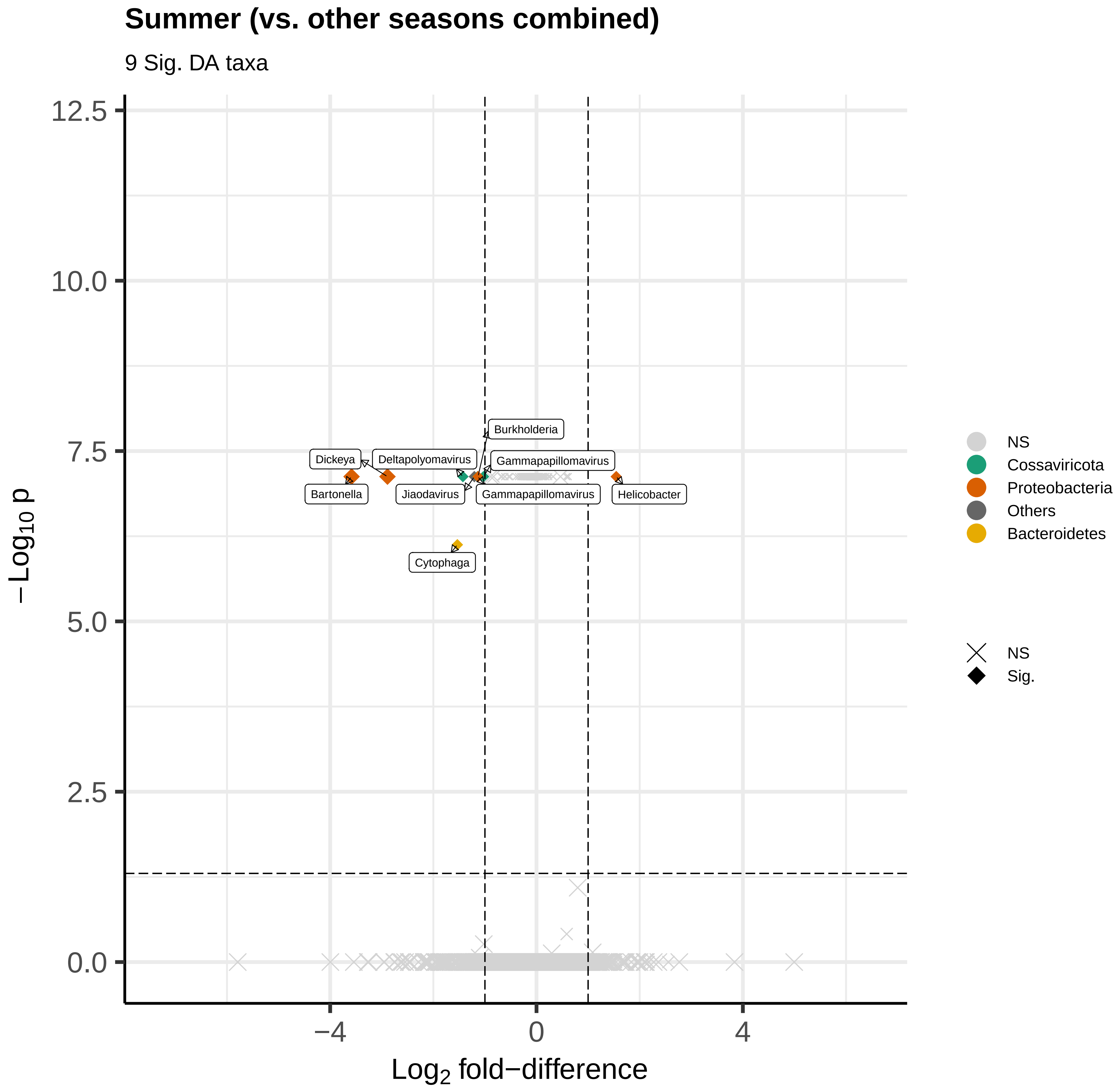

Summer (vs. other seasons combined)
9 Sig. DA taxa
12.5
10.0
7.5
5.0
2.5
0.0
−Log10 p
−4
0
4
Log2 fold−difference
Burkholderia
Dickeya
Deltapolyomavirus
Gammapapillomavirus
Bartonella
Jiaodavirus
Gammapapillomavirus
Helicobacter
Cytophaga
NS
Cossaviricota
Proteobacteria
Others
Bacteroidetes
NS
Sig.

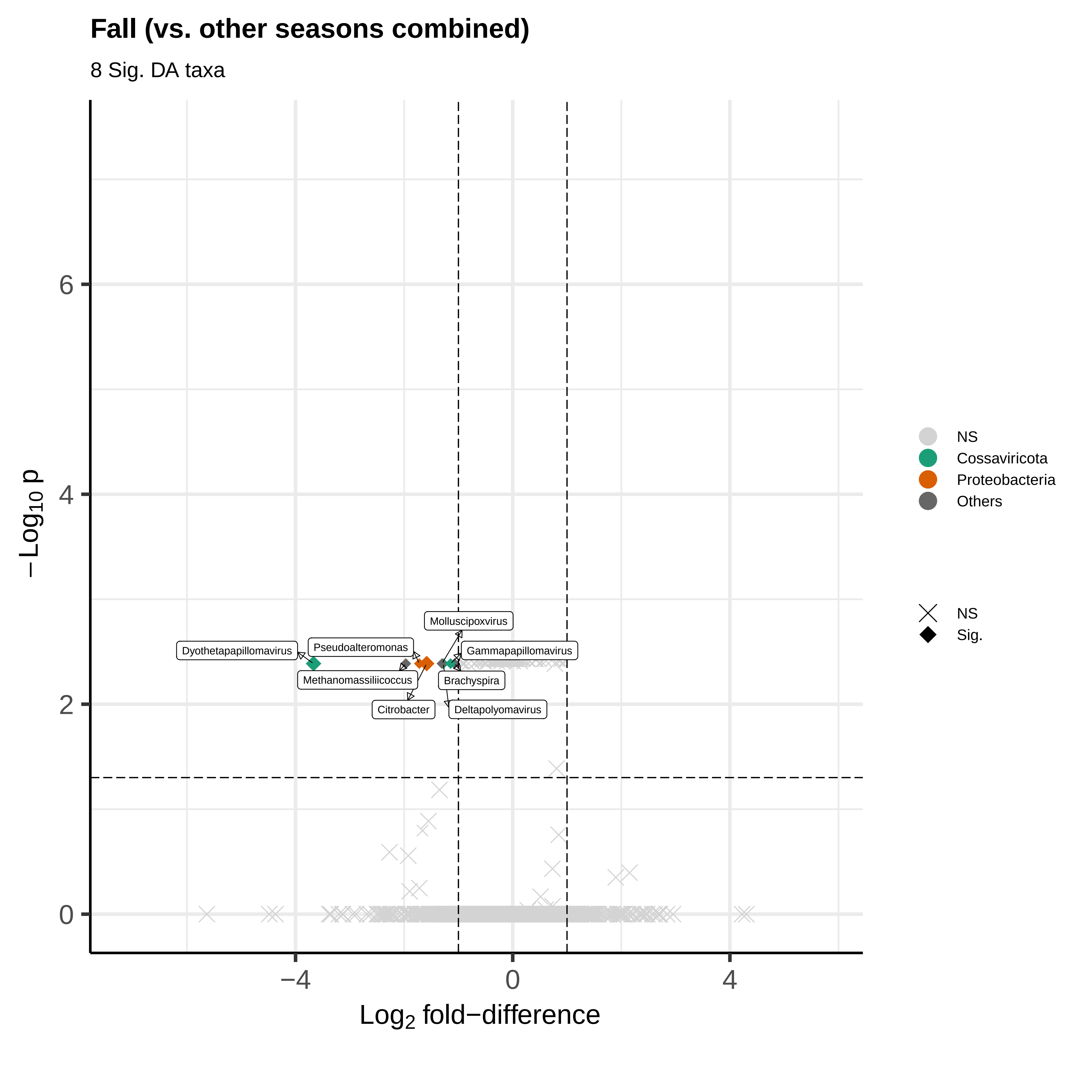

Fall (vs. other seasons combined)
8 Sig. DA taxa
−Log10 p
Log2 fold−difference
Dyothetapapillomavirus
Pseudoalteromonas
Methanomassiliicoccus
Citrobacter
Molluscipoxvirus
Gammapapillomavirus
Brachyspira
Deltapolyomavirus
NS
Cossaviricota
Proteobacteria
Others
NS
Sig.

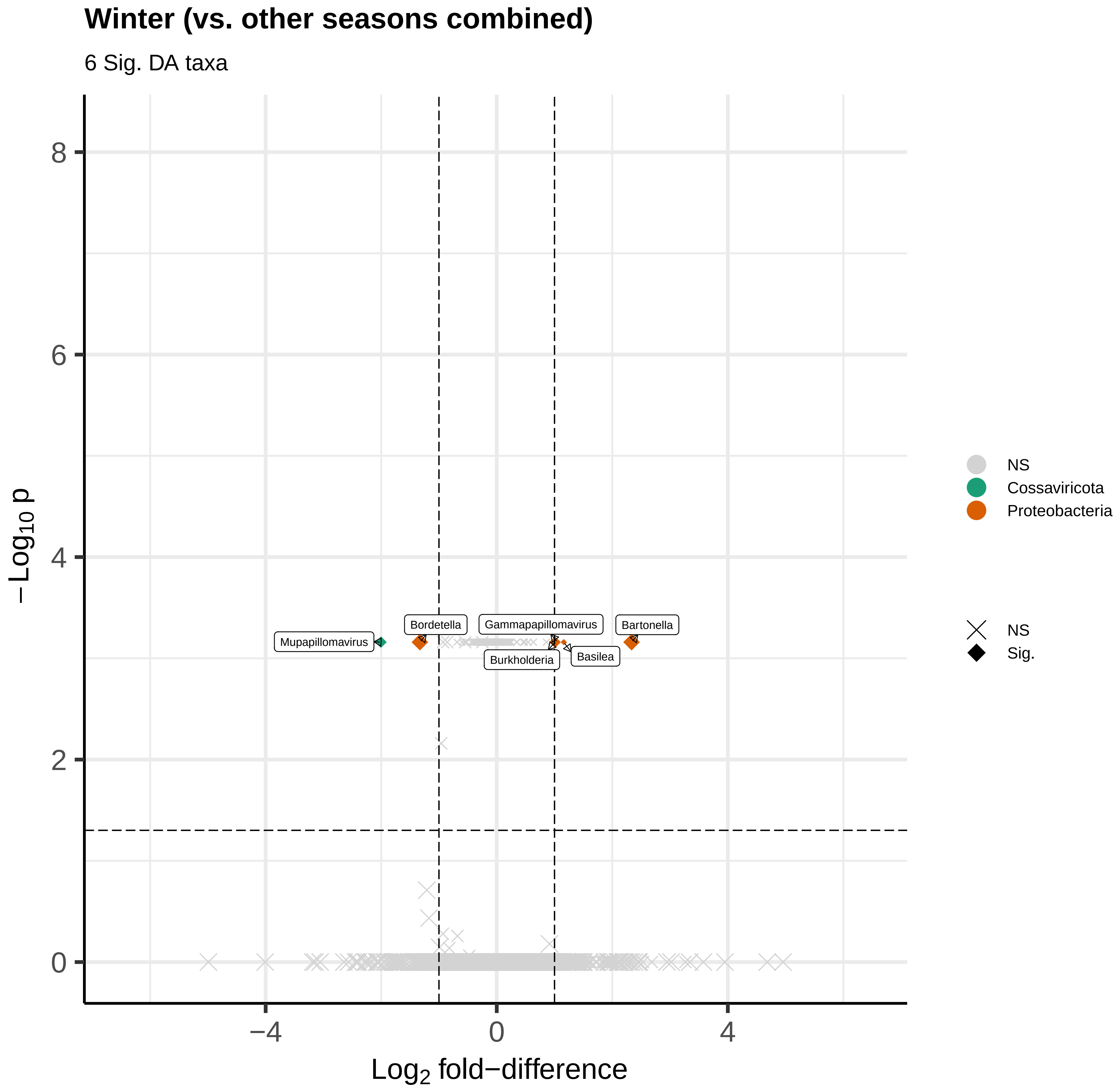

Winter (vs. other seasons combined)
6 Sig. DA taxa
8
6
4
2
0
−4
0
4
−Log10 p
Log2 fold−difference
Mupapillomavirus
Bordetella
Gammapapillomavirus
Bartonella
Burkholderia
Basilea
NS
Cossaviricota
Proteobacteria
NS
Sig.
