# Supplementary material for: Metagenomics reveals novel microbial signatures of farm exposures in house dust
Source: medRxiv. 2023 Apr 12:2023.04.07.23288301. Preprint. [Version 1] doi: 10.1101/2023.04.07.23288301 (PMC10120797; doi:10.1101/2023.04.07.23288301)
Supplement: Supplement 6 [file media-6.pdf]

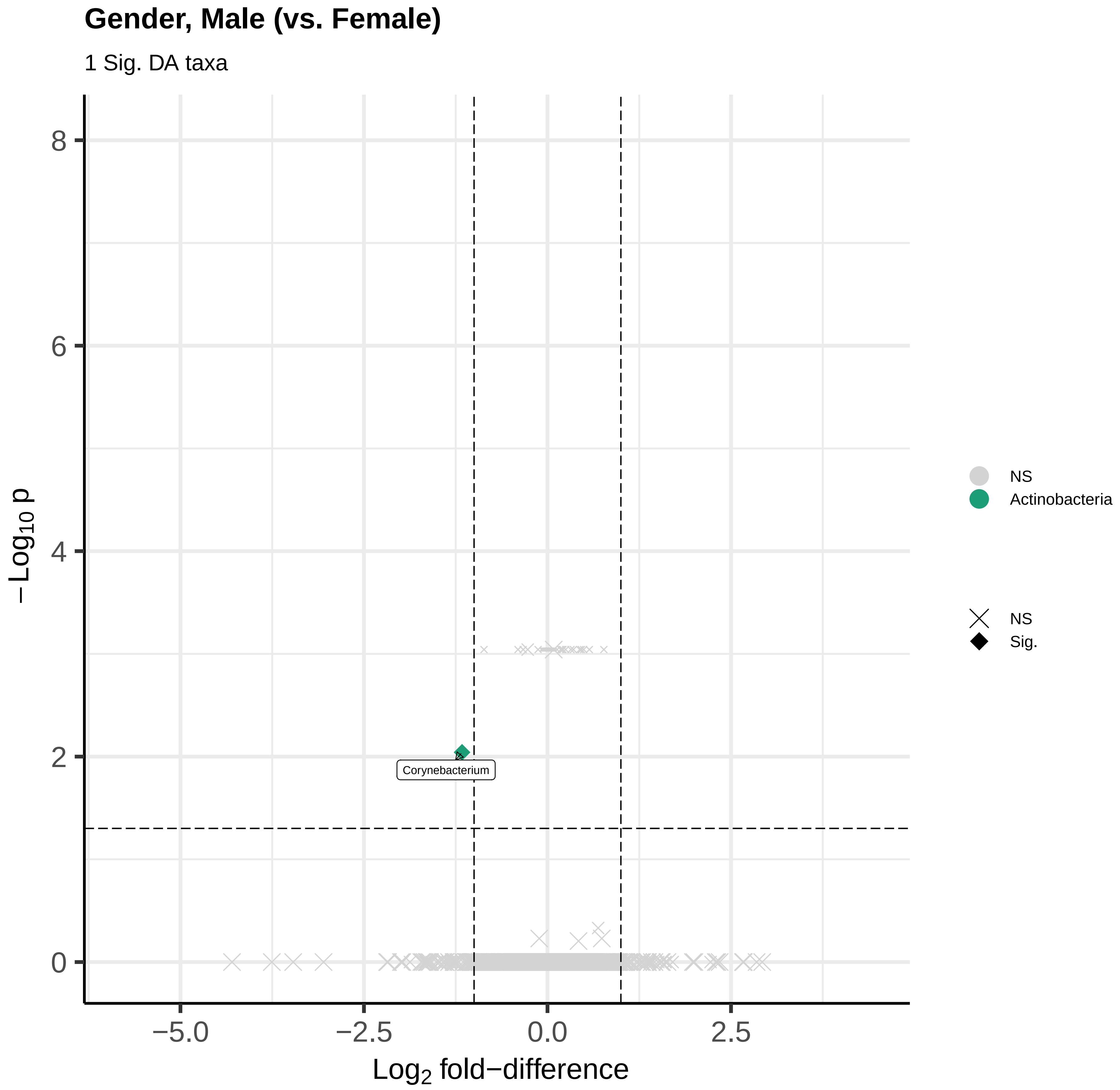

Gender, Male (vs. Female)
1 Sig. DA taxa
8
6
4
2
0
−Log10 p
Corynebacterium
−5.0
−2.5
0.0
2.5
Log2 fold−difference
NS
Actinobacteria
NS
Sig.

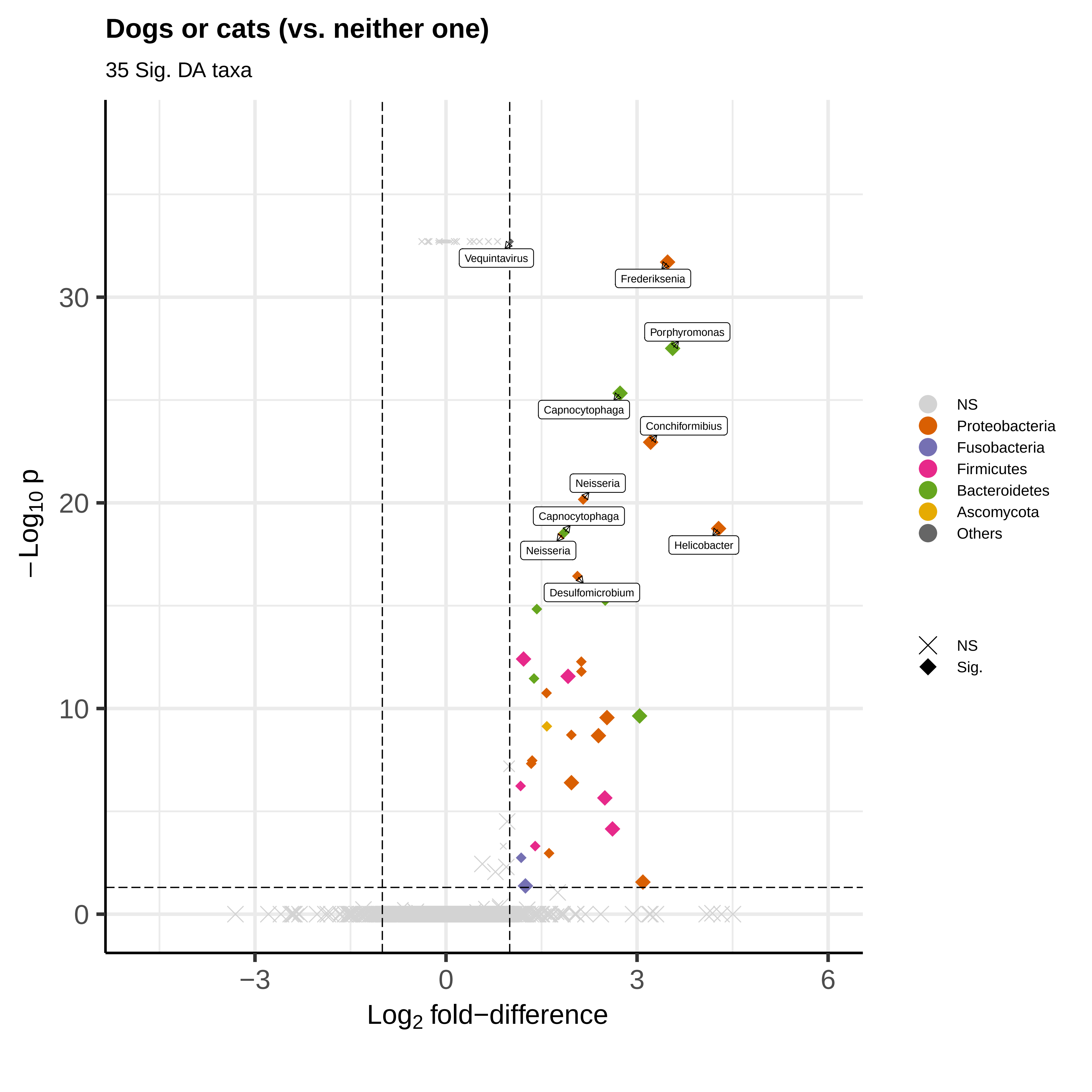
Dogs or cats (vs. neither one)
35 Sig. DA taxa
Vequintavirus
Frederiksenia
Porphyromonas
Capnocytophaga
Conchiformibius
Neisseria
Capnocytophaga
Neisseria
Helicobacter
Desulfomicrobium
−Log10 p
Log2 fold−difference
0
10
20
30
−3
0
3
6
NS
Proteobacteria
Fusobacteria
Firmicutes
Bacteroidetes
Ascomycota
Others
NS
Sig.

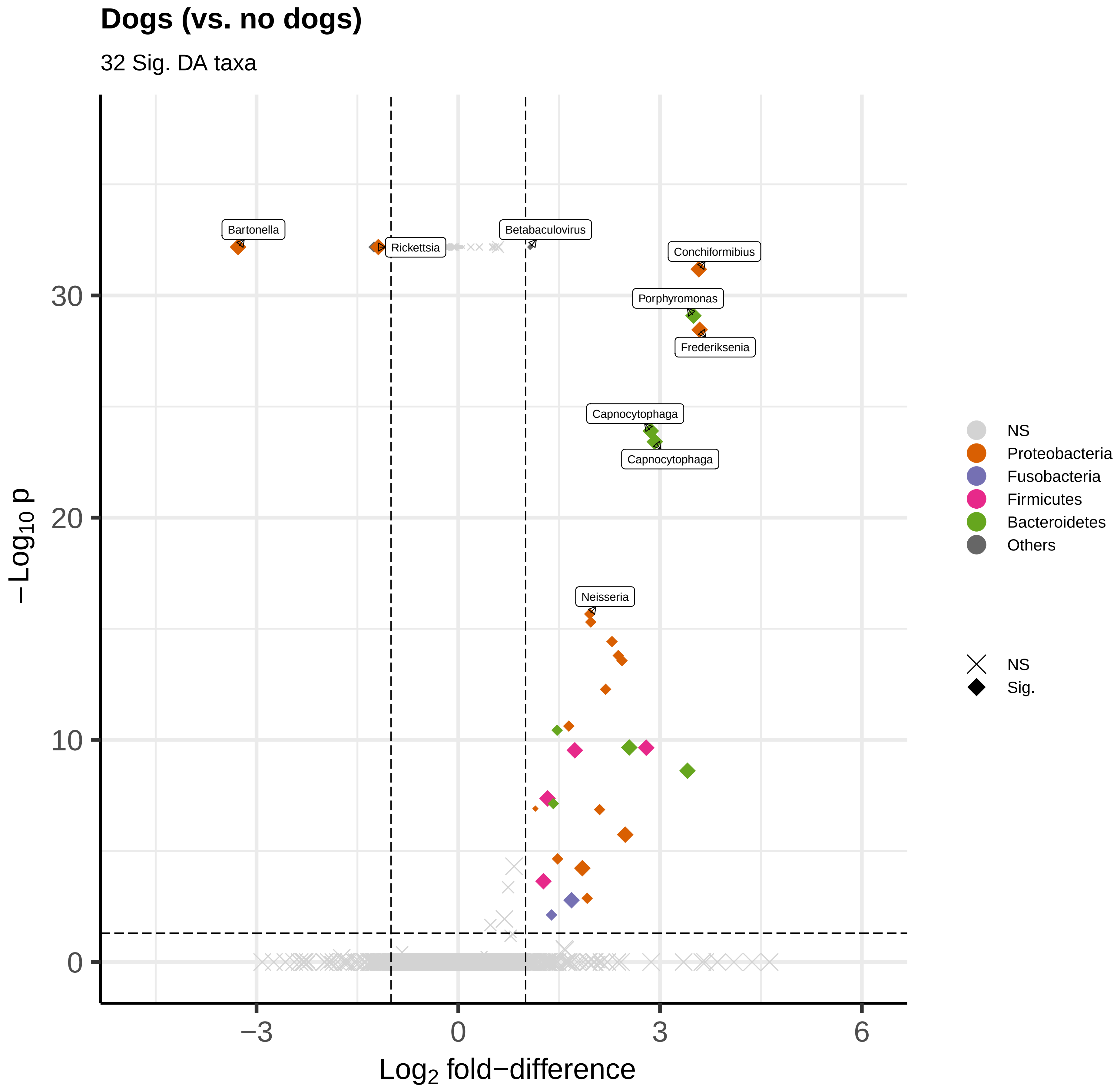

Dogs (vs. no dogs)
32 Sig. DA taxa
Bartonella
Rickettsia
Betabaculovirus
Conchiformibius
Porphyromonas
Frederiksenia
Capnocytophaga
Capnocytophaga
Neisseria
30
20
10
0
−3
0
3
6
−Log10 p
Log2 fold−difference
NS
Proteobacteria
Fusobacteria
Firmicutes
Bacteroidetes
Others
NS
Sig.

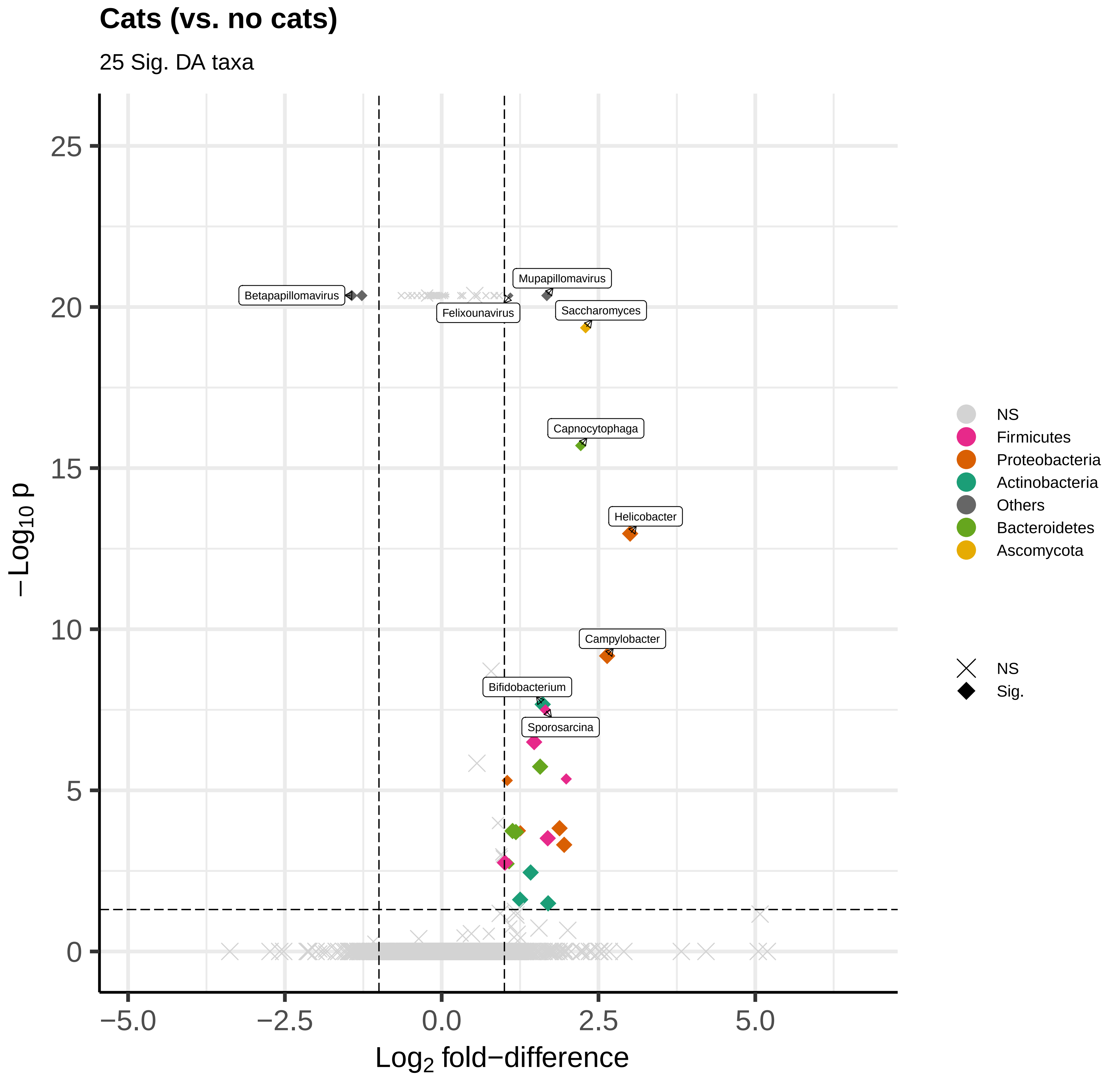
Cats (vs. no cats)
25 Sig. DA taxa
Betapapillomavirus
Mupapillomavirus
Felixounavirus
Saccharomyces
Capnocytophaga
Helicobacter
Campylobacter
Bifidobacterium
Sporosarcina
−Log10 p
Log2 fold−difference
25
20
15
10
5
0
−5.0
−2.5
0.0
2.5
5.0
NS
Firmicutes
Proteobacteria
Actinobacteria
Others
Bacteroidetes
Ascomycota
NS
Sig.

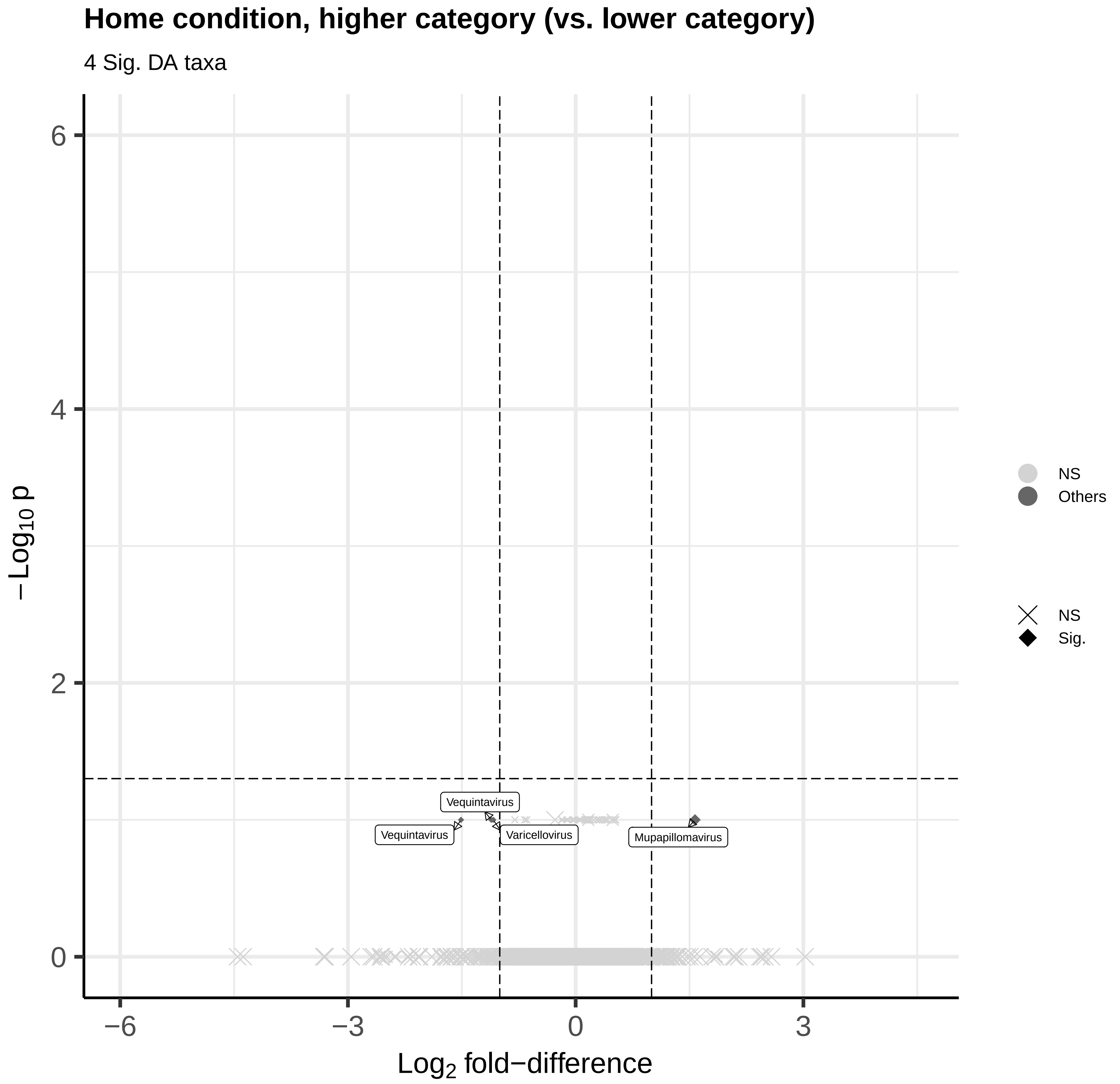
Home condition, higher category (vs. lower category)
4 Sig. DA taxa
−Log10 p
6
4
2
0
−6
−3
0
3
Log2 fold−difference
Vequintavirus
Vequintavirus
Varicellovirus
Mupapillomavirus
NS
Others
NS
Sig.

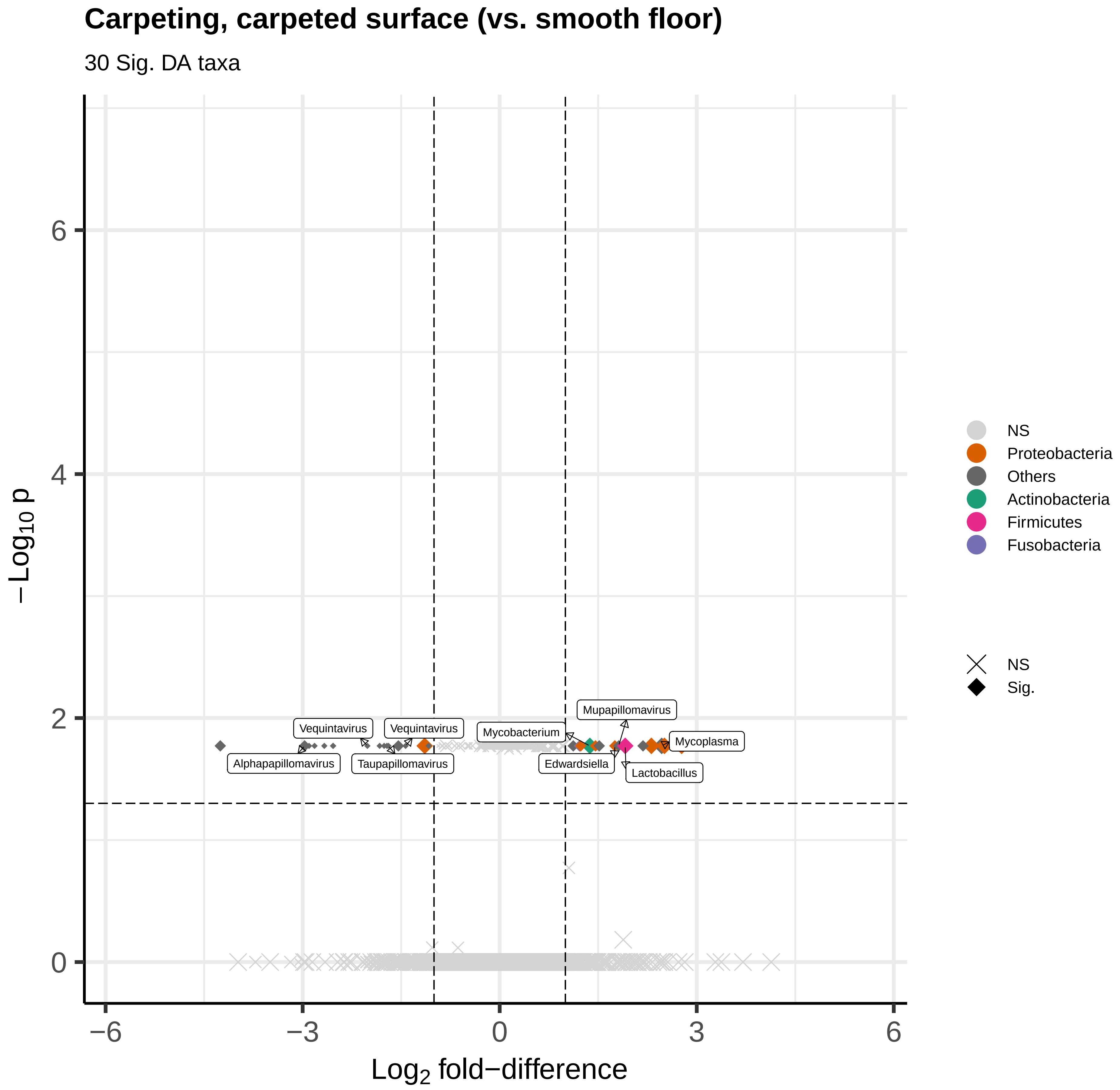
Carpeting, carpeted surface (vs. smooth floor)
30 Sig. DA taxa
−Log10 p
Log2 fold−difference
Vequintavirus
Vequintavirus
Mycobacterium
Mupapillomavirus
Mycoplasma
Alphapapillomavirus
Taupapillomavirus
Edwardsiella
Lactobacillus
NS
Proteobacteria
Others
Actinobacteria
Firmicutes
Fusobacteria
NS
Sig.

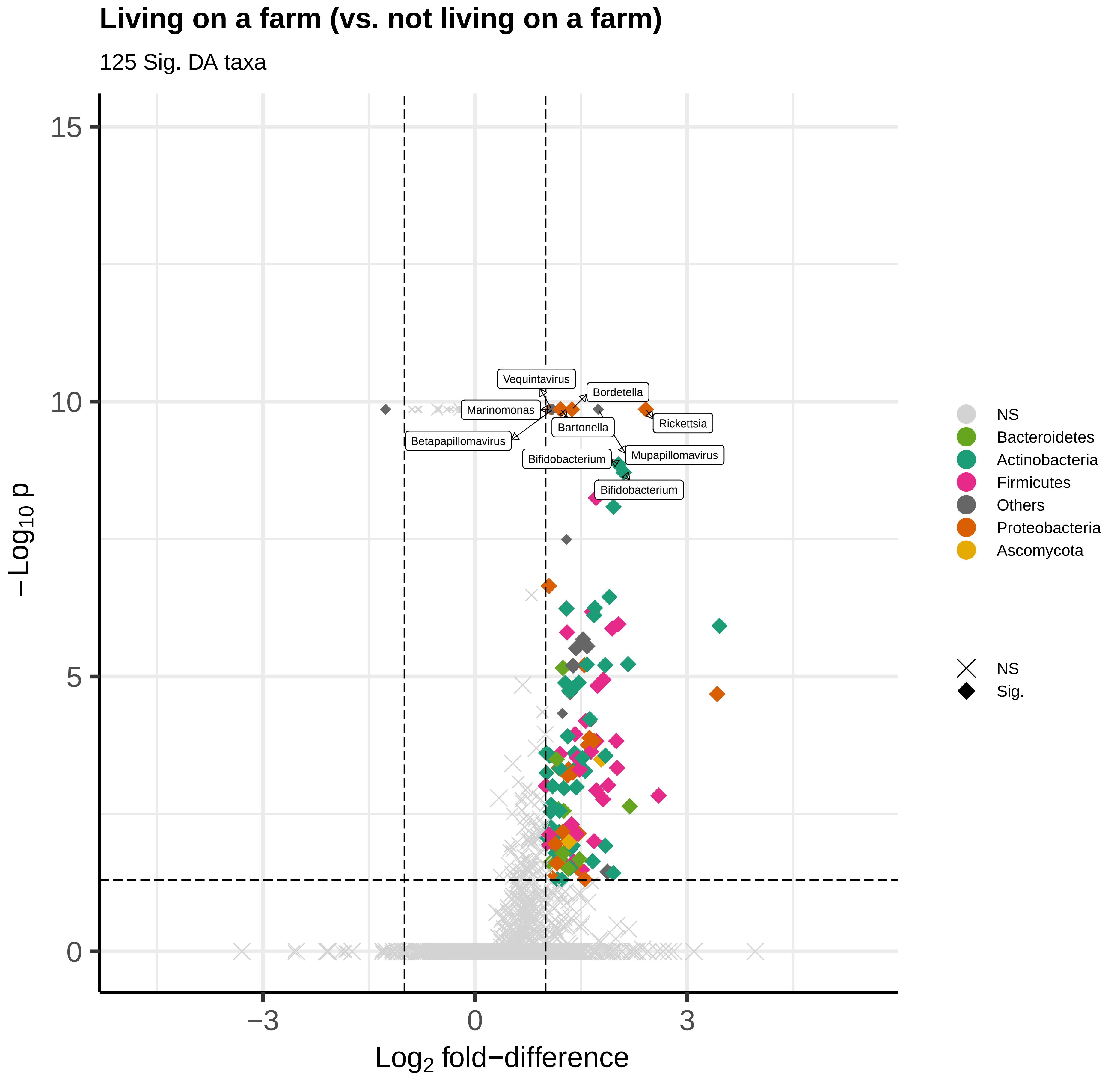
Living on a farm (vs. not living on a farm)
125 Sig. DA taxa
−Log10 p
Log2 fold−difference
15
10
5
0
−3
0
3
Vequintavirus
Bordetella
Marinomonas
Rickettsia
Bartonella
Betapapillomavirus
Mupapillomavirus
Bifidobacterium
Bifidobacterium
NS
Bacteroidetes
Actinobacteria
Firmicutes
Others
Proteobacteria
Ascomycota
NS
Sig.

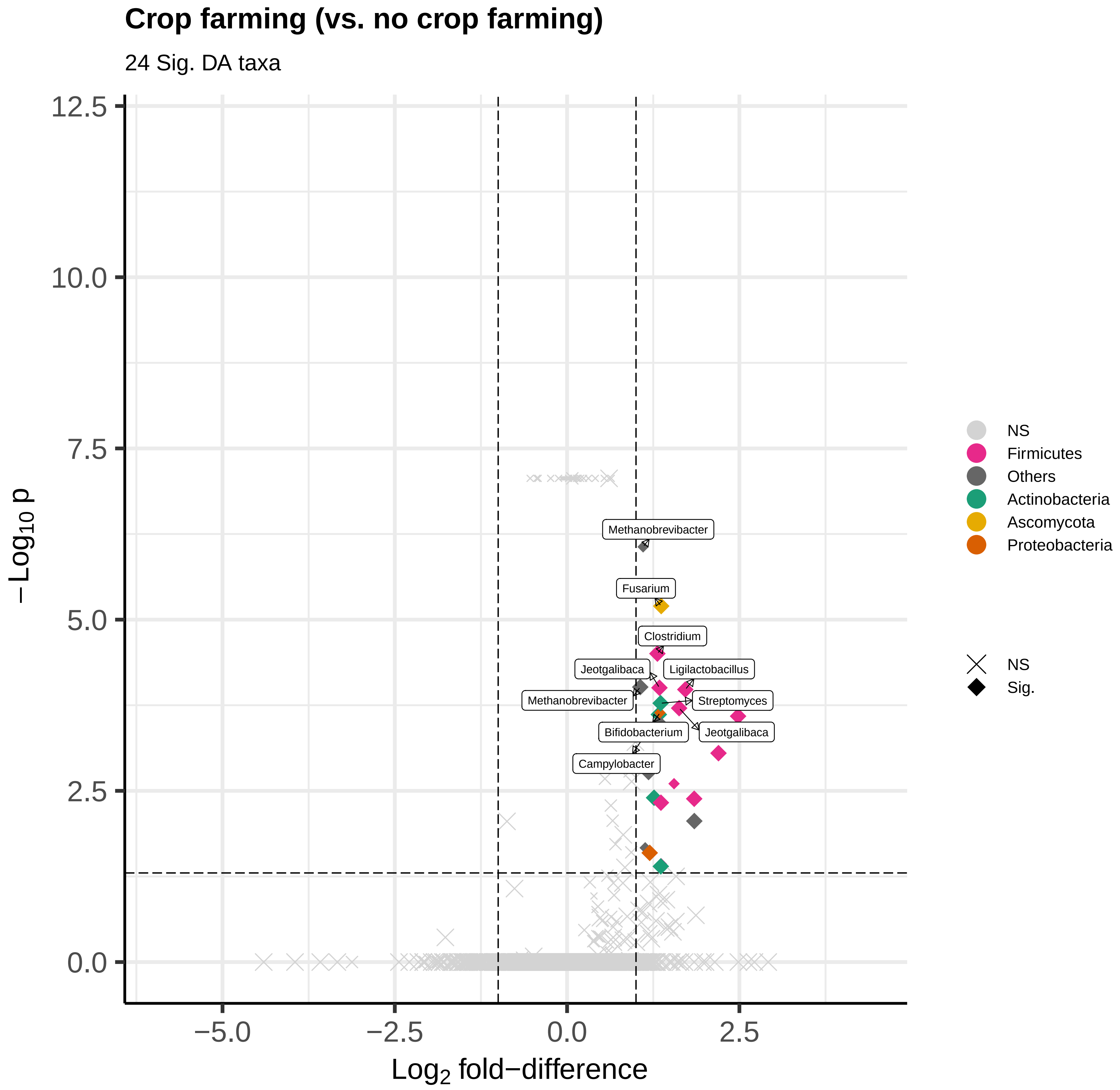
Crop farming (vs. no crop farming)
24 Sig. DA taxa
12.5
10.0
7.5
5.0
2.5
0.0
−Log10 p
−5.0
−2.5
0.0
2.5
Log2 fold−difference
Methanobrevibacter
Fusarium
Clostridium
Jeotgalibaca
Ligilactobacillus
Methanobrevibacter
Streptomyces
Bifidobacterium
Jeotgalibaca
Campylobacter
NS
Firmicutes
Others
Actinobacteria
Ascomycota
Proteobacteria
NS
Sig.

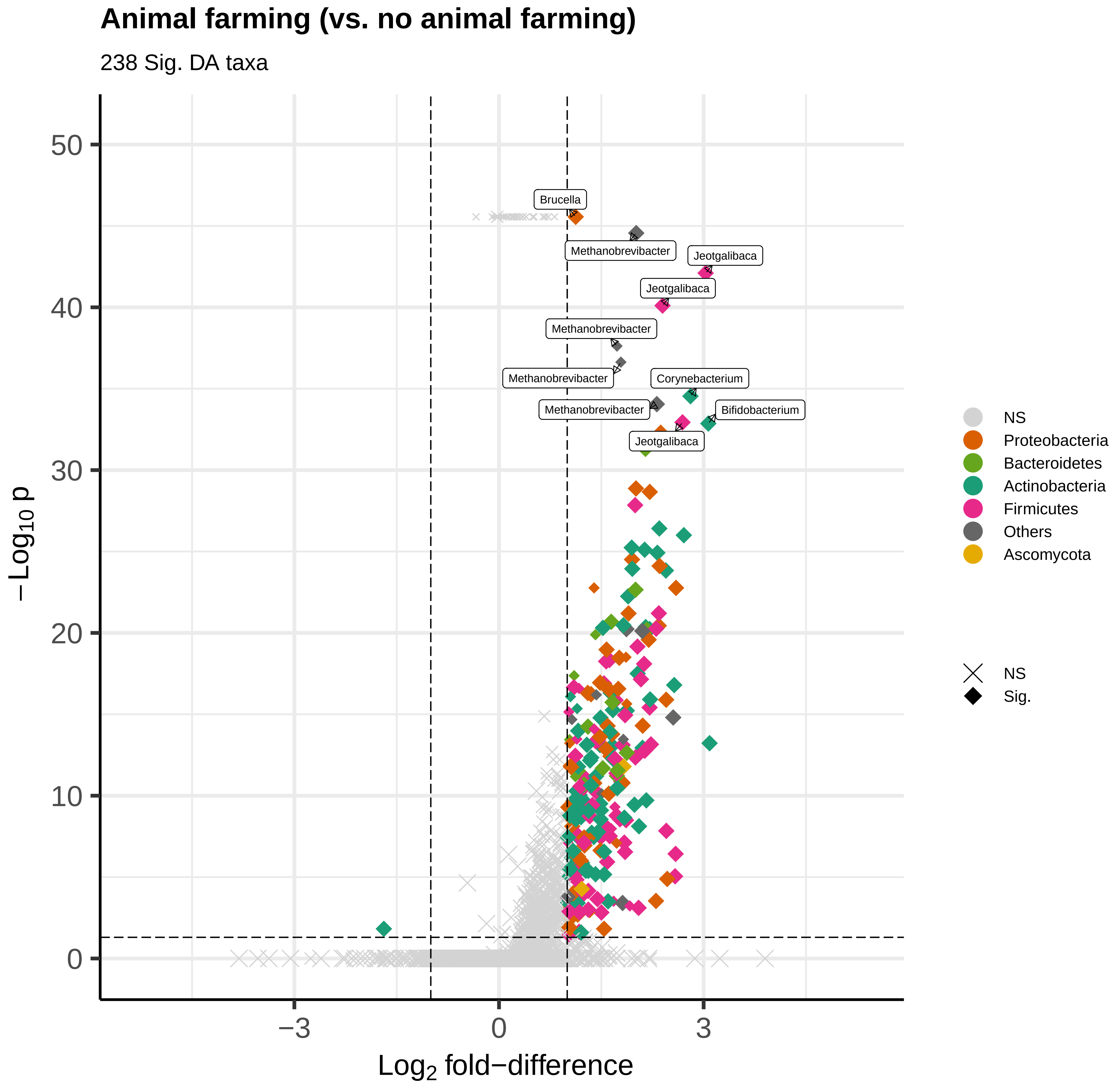
Animal farming (vs. no animal farming)
238 Sig. DA taxa
Brucella
Methanobrevibacter
Jeotgalibaca
Jeotgalibaca
Methanobrevibacter
Methanobrevibacter
Corynebacterium
Methanobrevibacter
Bifidobacterium
Jeotgalibaca
50
40
30
20
10
0
−3
0
3
−Log10 p
Log2 fold−difference
NS
Proteobacteria
Bacteroidetes
Actinobacteria
Firmicutes
Others
Ascomycota
NS
Sig.

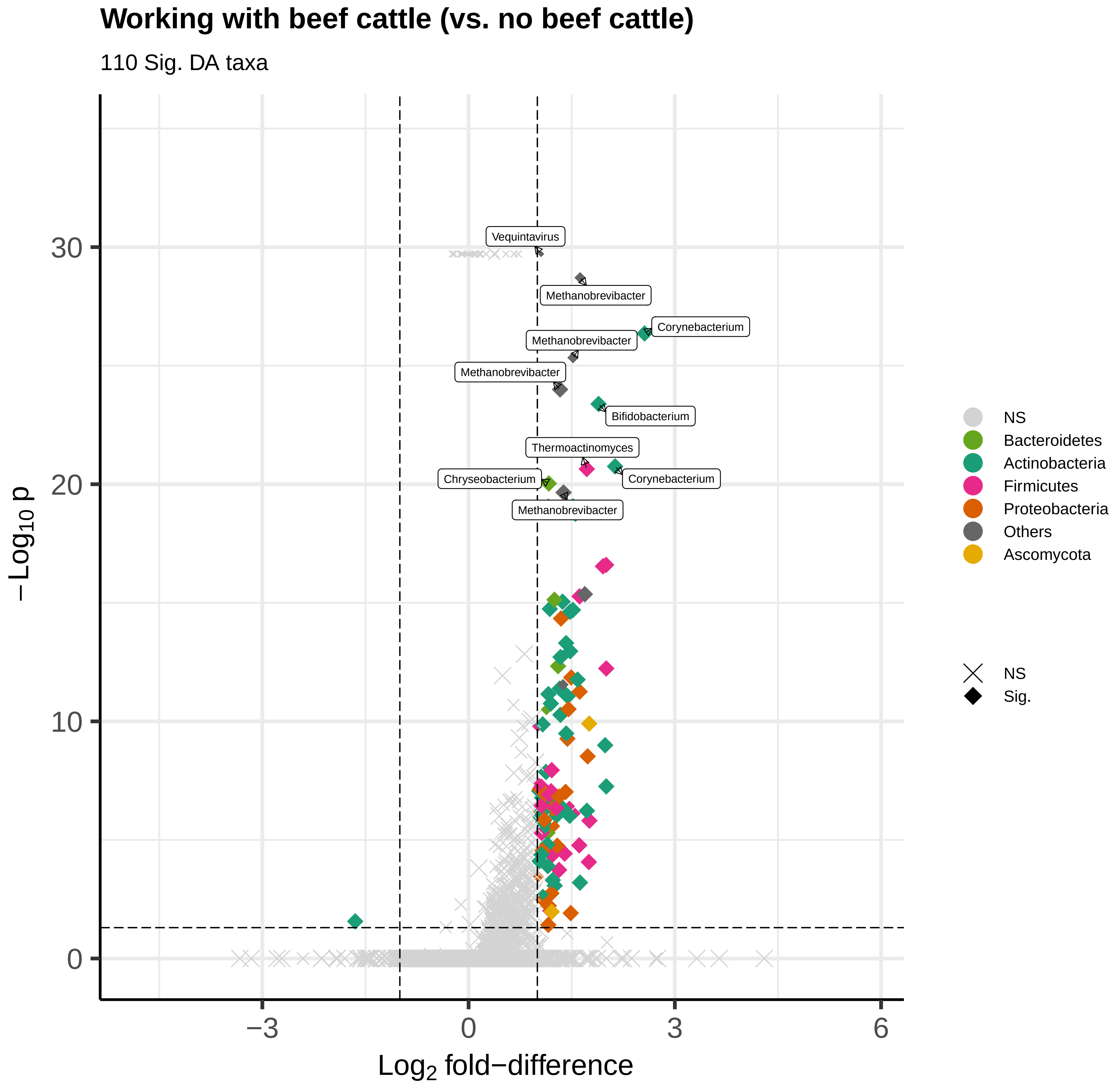

Working with beef cattle (vs. no beef cattle)
110 Sig. DA taxa
Vequintavirus
Methanobrevibacter
Corynebacterium
Methanobrevibacter
Methanobrevibacter
Bifidobacterium
Thermoactinomyces
Chryseobacterium
Corynebacterium
Methanobrevibacter
30
20
10
0
−3
0
3
6
Log2 fold−difference
−Log10 p
NS
Bacteroidetes
Actinobacteria
Firmicutes
Proteobacteria
Others
Ascomycota
NS
Sig.

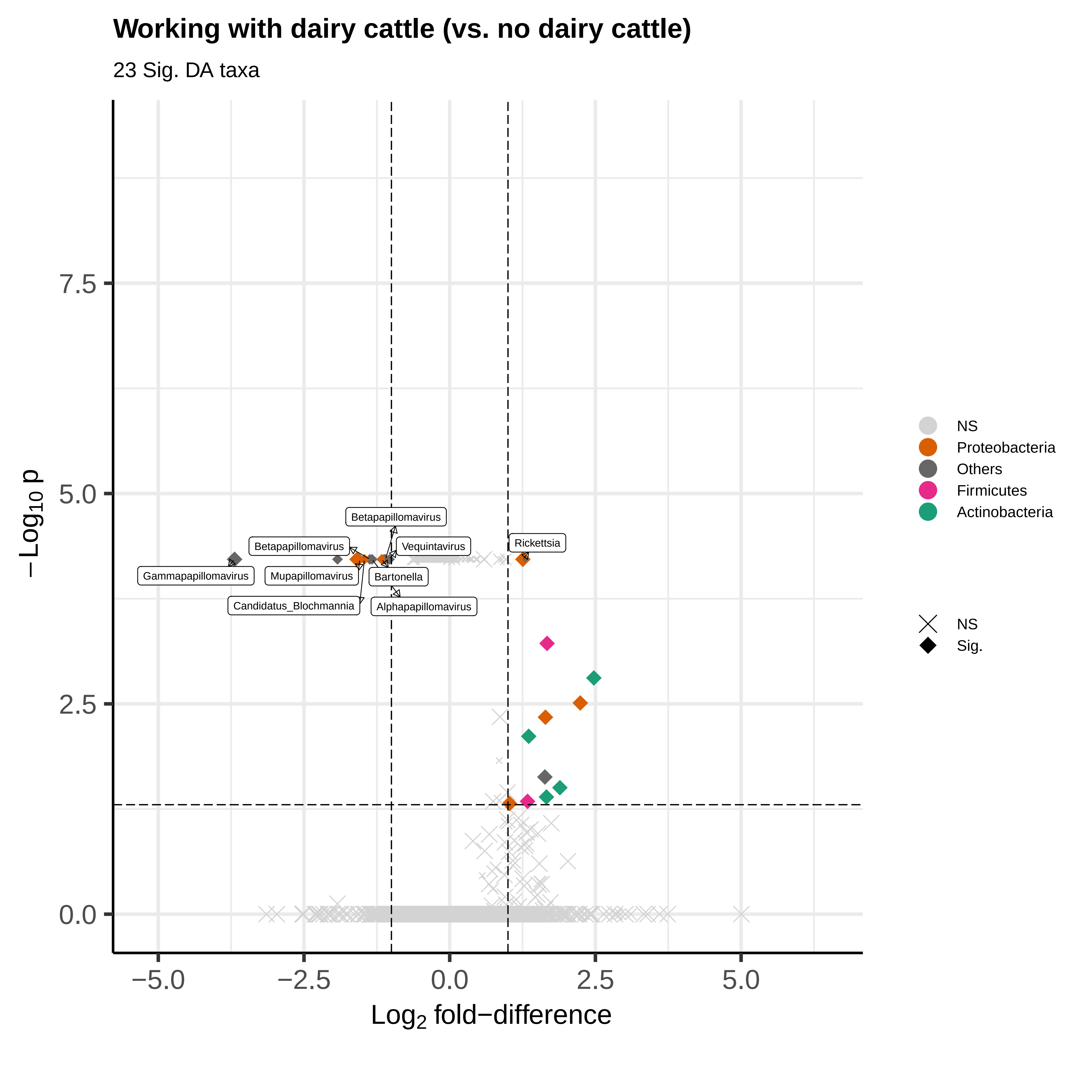
Working with dairy cattle (vs. no dairy cattle)
23 Sig. DA taxa
−Log10 p
7.5
5.0
2.5
0.0
−5.0
−2.5
0.0
2.5
5.0
Log2 fold−difference
Betapapillomavirus
Betapapillomavirus
Vequintavirus
Rickettsia
Gammapapillomavirus
Mupapillomavirus
Bartonella
Candidatus_Blochmannia
Alphapapillomavirus
NS
Proteobacteria
Others
Firmicutes
Actinobacteria
NS
Sig.

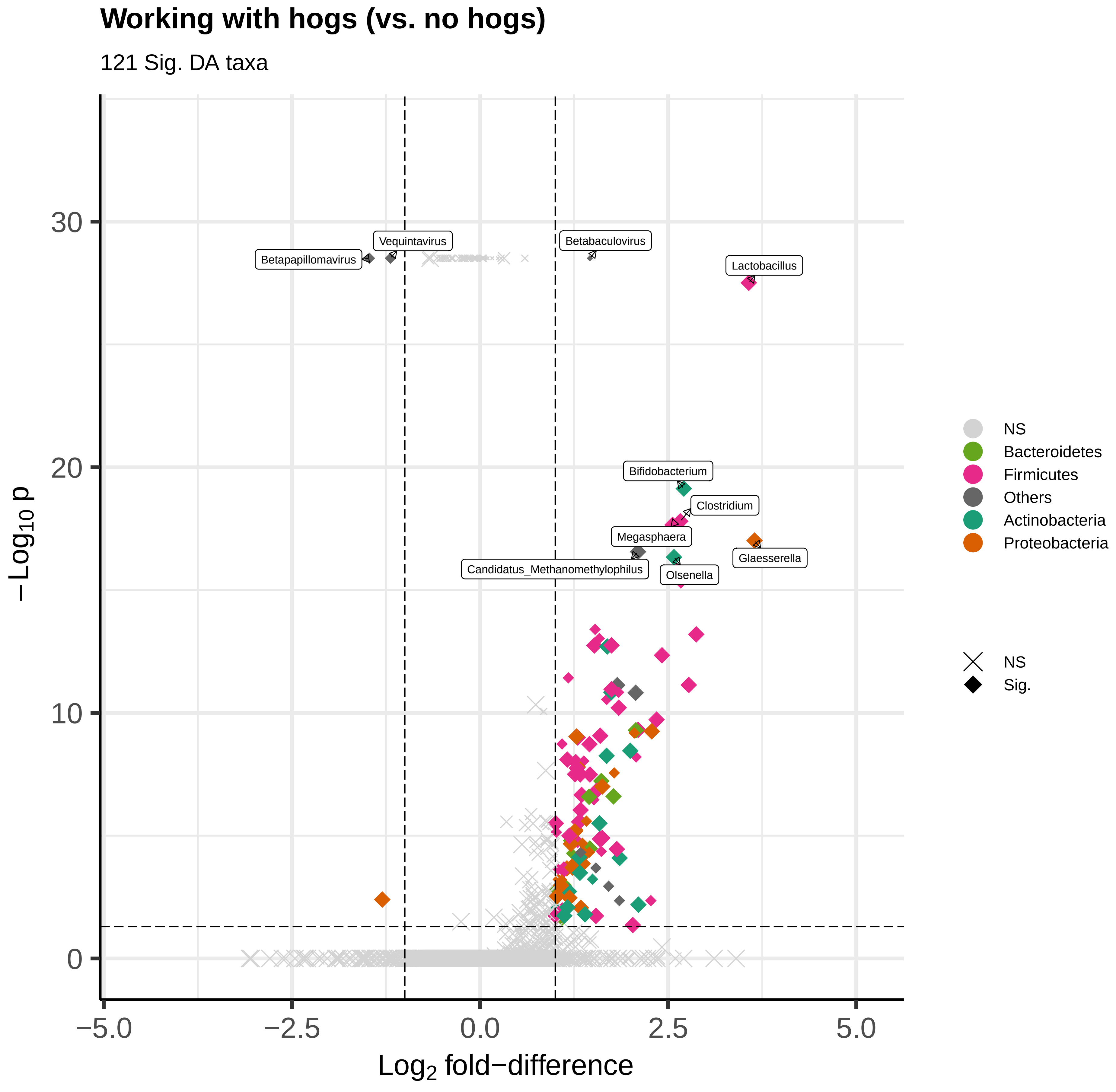

Working with hogs (vs. no hogs)
121 Sig. DA taxa
Betapapillomavirus
Vequintavirus
Betabaculovirus
Lactobacillus
Bifidobacterium
Clostridium
Megasphaera
Candidatus_Methanomethylophilus
Olsenella
Glaesserella
30
20
10
0
−5.0
−2.5
0.0
2.5
5.0
−Log10 p
Log2 fold−difference
NS
Bacteroidetes
Firmicutes
Others
Actinobacteria
Proteobacteria
NS
Sig.

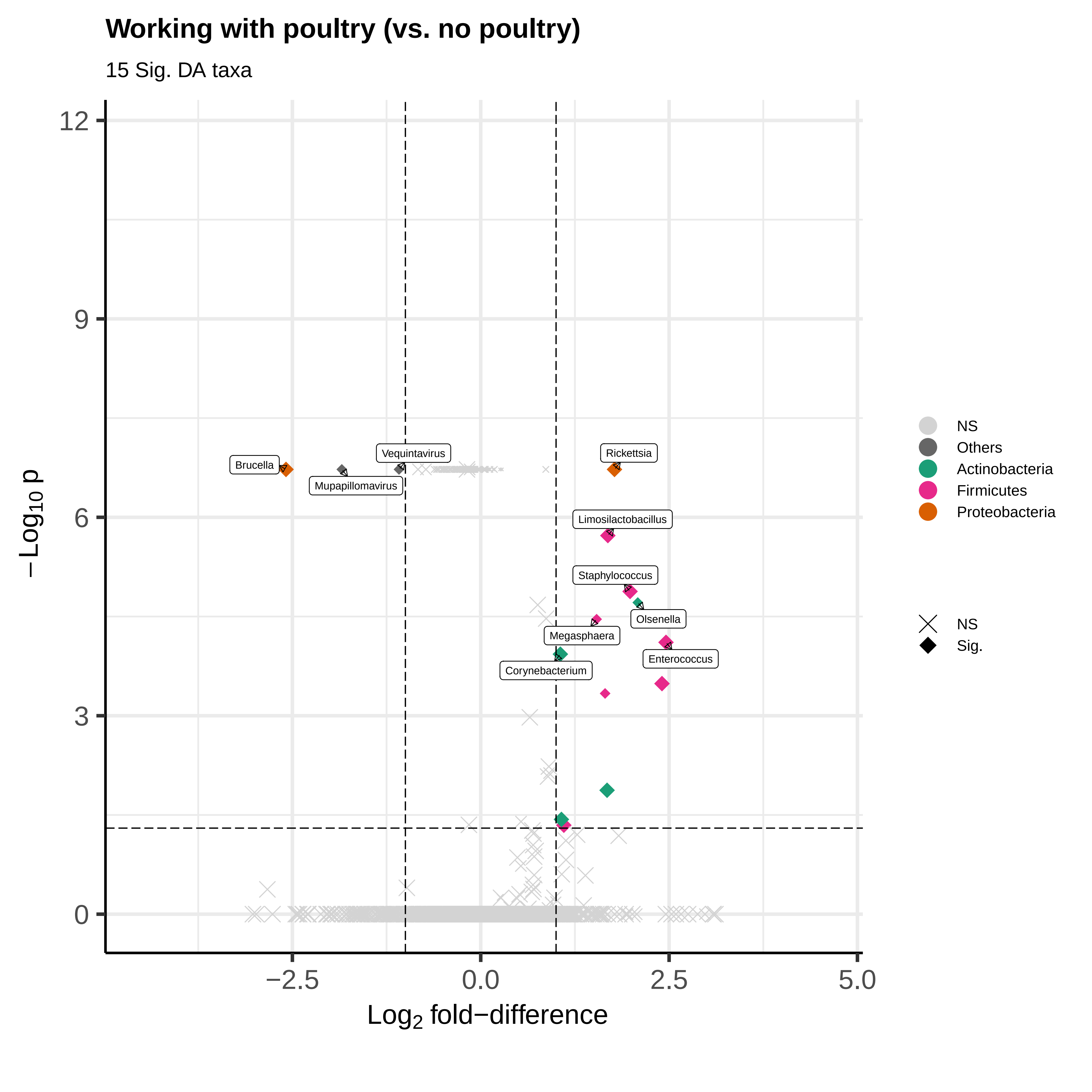
Working with poultry (vs. no poultry)
15 Sig. DA taxa
−Log10 p
Log2 fold−difference
12
9
6
3
0
−2.5
0.0
2.5
5.0
Brucella
Vequintavirus
Mupapillomavirus
Rickettsia
Limosilactobacillus
Staphylococcus
Olsenella
Megasphaera
Enterococcus
Corynebacterium
NS
Others
Actinobacteria
Firmicutes
Proteobacteria
NS
Sig.

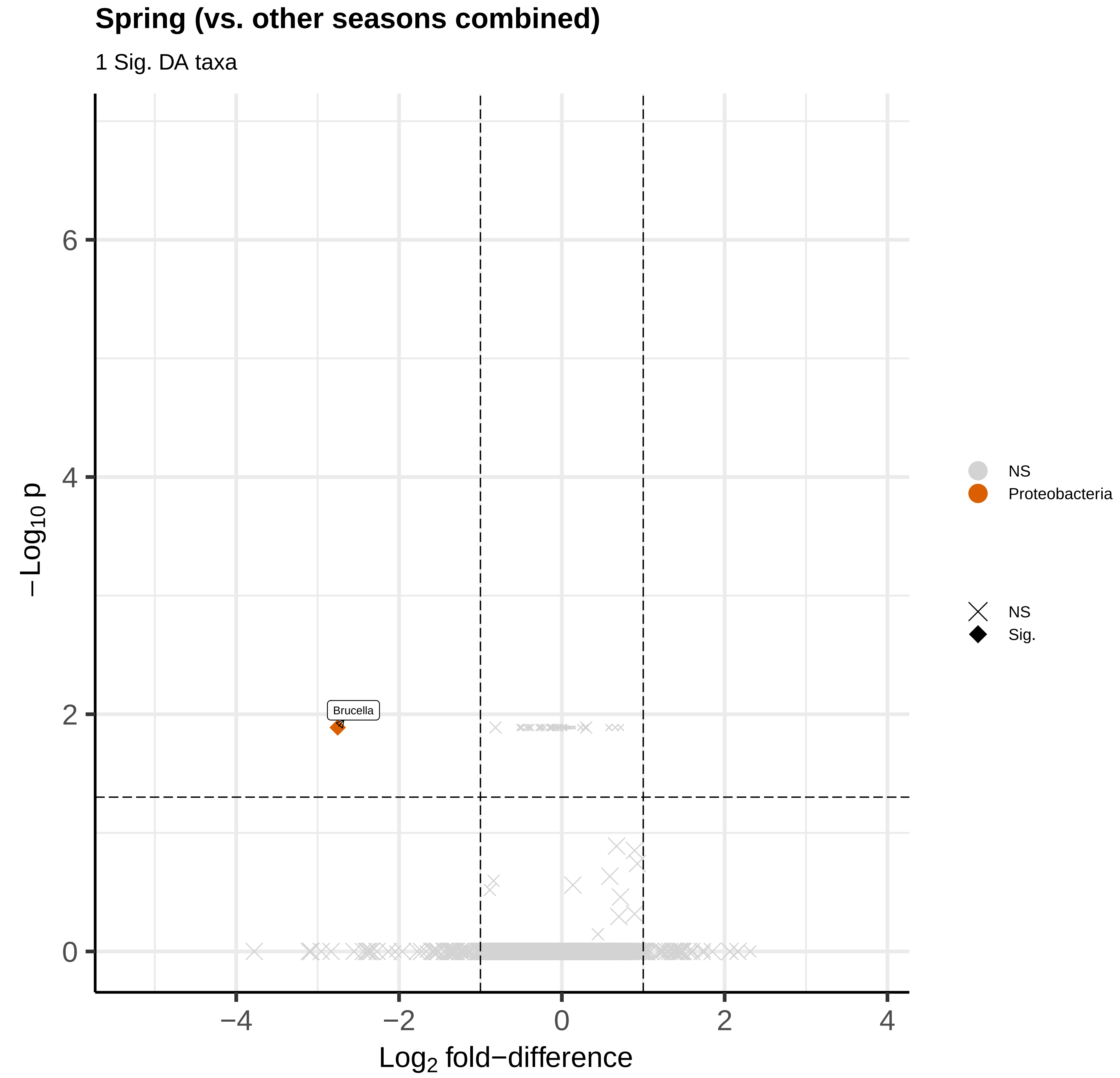
Spring (vs. other seasons combined)
1 Sig. DA taxa
Brucella
6
4
2
0
−Log10 p
−4
−2
0
2
4
Log2 fold−difference
NS
Proteobacteria
NS
Sig.

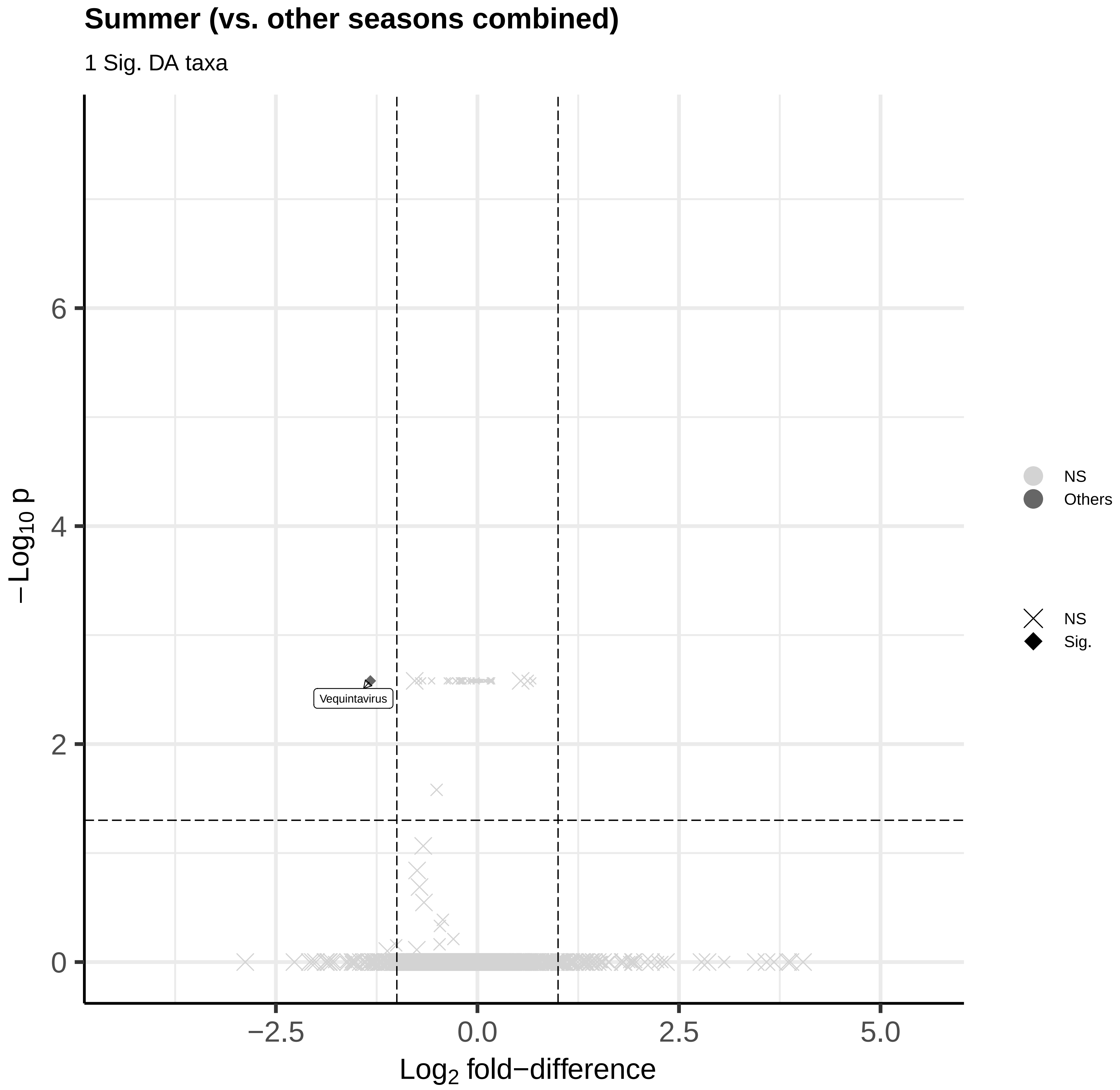
Summer (vs. other seasons combined)
1 Sig. DA taxa
Vequintavirus
6
4
2
0
−2.5
0.0
2.5
5.0
−Log10 p
Log2 fold−difference
NS
Others
NS
Sig.

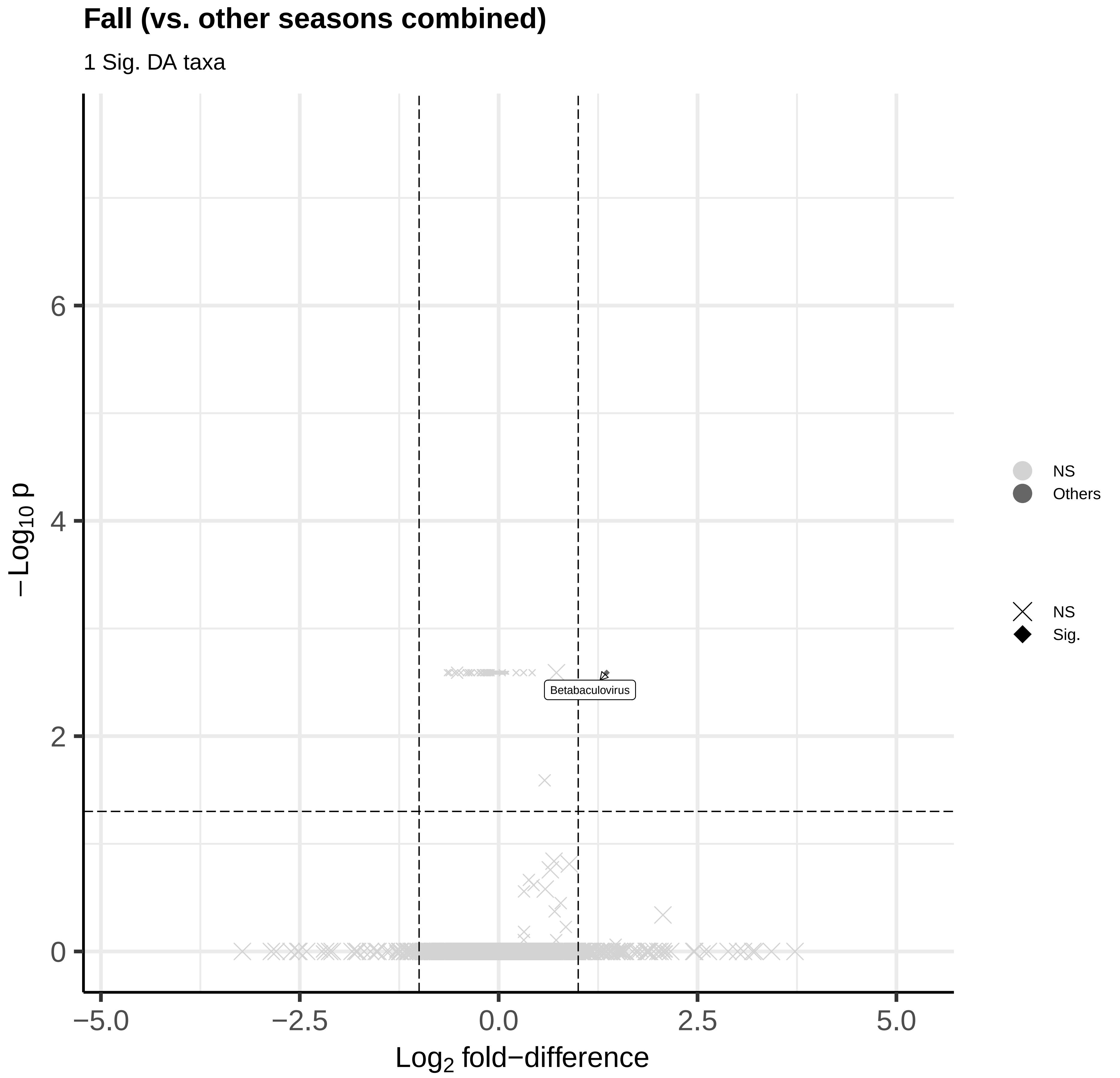

Fall (vs. other seasons combined)
1 Sig. DA taxa
−Log10 p
6
4
2
0
−5.0
−2.5
0.0
2.5
5.0
Log2 fold−difference
Betabaculovirus
NS
Others
NS
Sig.

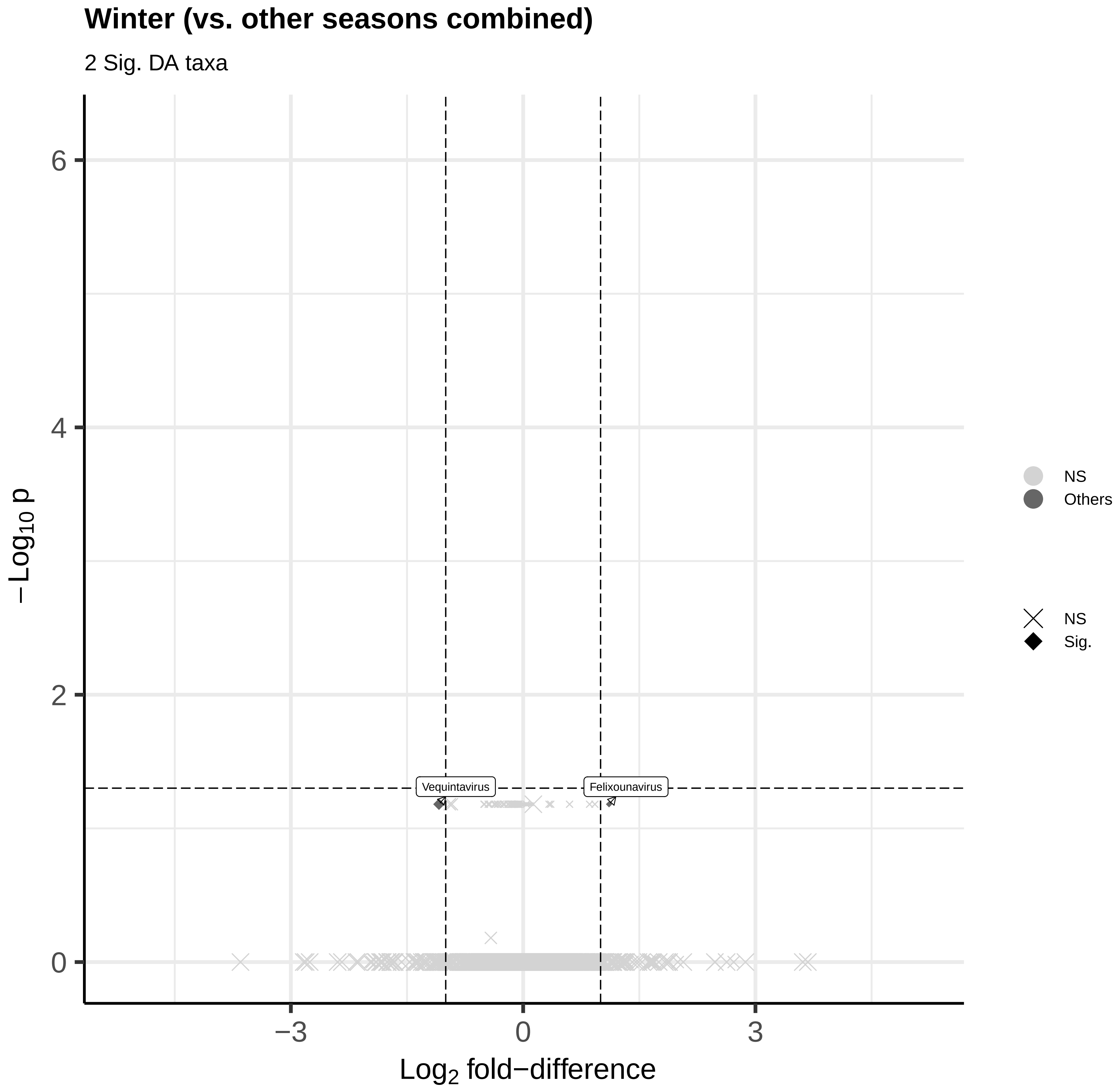
Winter (vs. other seasons combined)
2 Sig. DA taxa
6
4
2
0
−3
0
3
Log2 fold−difference
−Log10 p
Vequintavirus
Felixounavirus
NS
Others
NS
Sig.
